# Tomato SlERF26 enhances drought tolerance by regulating SlPIP2;4-mediated ROS homeostasis

**DOI:** 10.1093/hr/uhag215

**Published:** 2026-06-02

**Authors:** Yaling Wang, Xiaomeng Hu, Jiayan Yang, Yongjie Mo, Huifang Hou, Yin Ding, Wenrui Gong, Pan Zeng, Chunyu Shang, Xingguo Zhang, Yu Pan, Lang Wu, Hongyuan Song, Nan Hu, Dongyang Dai, Md Mahmudul Hasan, Jinhua Li

**Affiliations:** Key Laboratory of Agricultural Biosafety and Green Production of Upper Yangtze River (Ministry of Education), College of Horticulture and Landscape Architecture, Southwest University, No.2 Tiansheng Road, Beibei, Chongqing 400715, China; Key Laboratory of Agricultural Biosafety and Green Production of Upper Yangtze River (Ministry of Education), College of Horticulture and Landscape Architecture, Southwest University, No.2 Tiansheng Road, Beibei, Chongqing 400715, China; Key Laboratory of Agricultural Biosafety and Green Production of Upper Yangtze River (Ministry of Education), College of Horticulture and Landscape Architecture, Southwest University, No.2 Tiansheng Road, Beibei, Chongqing 400715, China; Key Laboratory of Agricultural Biosafety and Green Production of Upper Yangtze River (Ministry of Education), College of Horticulture and Landscape Architecture, Southwest University, No.2 Tiansheng Road, Beibei, Chongqing 400715, China; Key Laboratory of Agricultural Biosafety and Green Production of Upper Yangtze River (Ministry of Education), College of Horticulture and Landscape Architecture, Southwest University, No.2 Tiansheng Road, Beibei, Chongqing 400715, China; Key Laboratory of Agricultural Biosafety and Green Production of Upper Yangtze River (Ministry of Education), College of Horticulture and Landscape Architecture, Southwest University, No.2 Tiansheng Road, Beibei, Chongqing 400715, China; Key Laboratory of Agricultural Biosafety and Green Production of Upper Yangtze River (Ministry of Education), College of Horticulture and Landscape Architecture, Southwest University, No.2 Tiansheng Road, Beibei, Chongqing 400715, China; Key Laboratory of Agricultural Biosafety and Green Production of Upper Yangtze River (Ministry of Education), College of Horticulture and Landscape Architecture, Southwest University, No.2 Tiansheng Road, Beibei, Chongqing 400715, China; Key Laboratory of Agricultural Biosafety and Green Production of Upper Yangtze River (Ministry of Education), College of Horticulture and Landscape Architecture, Southwest University, No.2 Tiansheng Road, Beibei, Chongqing 400715, China; Key Laboratory of Agricultural Biosafety and Green Production of Upper Yangtze River (Ministry of Education), College of Horticulture and Landscape Architecture, Southwest University, No.2 Tiansheng Road, Beibei, Chongqing 400715, China; Key Laboratory of Agricultural Biosafety and Green Production of Upper Yangtze River (Ministry of Education), College of Horticulture and Landscape Architecture, Southwest University, No.2 Tiansheng Road, Beibei, Chongqing 400715, China; Key Laboratory of Agricultural Biosafety and Green Production of Upper Yangtze River (Ministry of Education), College of Horticulture and Landscape Architecture, Southwest University, No.2 Tiansheng Road, Beibei, Chongqing 400715, China; Key Laboratory of Agricultural Biosafety and Green Production of Upper Yangtze River (Ministry of Education), College of Horticulture and Landscape Architecture, Southwest University, No.2 Tiansheng Road, Beibei, Chongqing 400715, China; College of Horticulture and Forestry, Xinjiang Production &Construction Corps Key Laboratory of Protected Agriculture, Tarim University, Alar, Xinjiang 843300, China; College of Horticulture and Forestry, Xinjiang Production &Construction Corps Key Laboratory of Protected Agriculture, Tarim University, Alar, Xinjiang 843300, China; Biotechnology and Germplasm Resources Institute, Yunnan Academy of Agricultural Sciences, Kunming 650205, China; Key Laboratory of Agricultural Biosafety and Green Production of Upper Yangtze River (Ministry of Education), College of Horticulture and Landscape Architecture, Southwest University, No.2 Tiansheng Road, Beibei, Chongqing 400715, China

## Abstract

Adverse environmental conditions severely limit plant growth and productivity, especially under an increasingly frequent and severe drought. Cultivated tomato is highly sensitive to drought, while wild tomato species exhibit remarkable drought tolerance. In this study, we identified *SlERF26* as a drought-responsive gene that was differentially expressed between drought-tolerant *Solanum pennellii* introgression lines and the cultivated tomato background through combined introgression-line and transcriptomic analyses. *SlERF26* encodes an AP2/ERF (APETALA2/Ethylene Response Factor) transcription factor. Under drought stress, overexpression of *SlERF26* reduced the accumulation of reactive oxygen species (ROS), including H₂O₂ and O₂^−^, which is consistent with its positive role in regulating drought tolerance. Transcriptome profiling further indicated that *SlERF26* regulated a series of drought-responsive genes involved in membrane transport and ROS scavenging, such as aquaporin and peroxidases. Specifically, SlERF26 directly bound to the promoter of *SlPIP2;4*, an aquaporin gene, and activated its transcription. The *slpip2;4* mutant accumulated higher ROS levels and exhibited significantly reduced drought tolerance. Moreover, analysis of *SlERF26* OE × *slpip2;4* hybrid plants confirms that *SlERF26* acts upstream of *SlPIP2;4* in drought tolerance. Consistent with this genetic relationship, biochemical quantification and histochemical staining demonstrated that SlERF26 and SlPIP2;4 act together to maintain ROS homeostasis during drought stress. Collectively, our findings define a SlERF26–SlPIP2;4 regulatory module that enhances drought tolerance in tomato by modulating aquaporin expression and ROS homeostasis.

## Introduction

Plants are stationary and constantly threatened by various environmental stresses, such as drought, salinity, extreme temperatures, metal toxicity, and light stress. Among them, drought is considered a major limiting factor that severely affects plant development, crop yield, and global food security [1–3]. More critically, drought stress disrupts cellular osmotic balance, compromises cell membrane integrity, and inhibits the activities of metabolic enzymes [4]. As a result, prolonged or severe drought can substantially restrict plant growth and development, leading to significant yield reductions or even plant mortality. To enhance drought tolerance, plants have developed sophisticated physiological and biochemical adaptive strategies, including stomatal regulation, root architecture remodeling, antioxidant defense systems, and phytohormone-mediated signaling pathways [5–10]. Nevertheless, enhancing plant adaptability to drought remains a major challenge, especially under ongoing climate change and rising demands for agricultural water resources. Genetic engineering and advanced breeding strategies offer promising approaches for rapidly and precisely improving drought tolerance, thereby minimizing crop yield losses. Thus, identifying key genes involved in drought tolerance and deciphering their regulatory networks are crucial steps toward developing drought-resilient tomato varieties.

Transcription factors (TFs) are key regulators of gene transcription, modulating the expression of downstream genes through sequence-specific DNA binding and interactions with regulatory cofactors. Extensive research has demonstrated that TFs serve as central regulators in plant responses to drought stress, with key contributions from the APETALA2/ethylene response factor (AP2/ERF) [11], NAC [12], and BBX [13] families. The AP2/ERF family represents one of the largest TF groups in plants [14]. AP2/ERF proteins possess one or two AP2/ERF domains, each comprising ~60–70 amino acids, which are essential for DNA binding. This family comprises: ERF, AP2, RAV (related to ABI3/VP1), and DREB (dehydration-responsive element-binding protein) [15, 16] The ERF subfamily contains a single AP2/ERF domain and can be further divided into two major groups: the CBF/DREB and the ERF subfamilies [14, 15]. CBF/DREB proteins bind to the dehydration-responsive element/C-repeat (DRE/CRT), featuring a core A/GCCGAC sequence, thereby enhancing tolerance to abiotic stresses. In contrast, ERF proteins specifically bind to the AGCCGCC motif, also referred to as the GCC-box or ethylene-responsive element (ERE), and contribute primarily to resistance against biotic stresses [15, 17]. Notably, many AP2/ERF TFs are capable of recognizing both DRE/CRT and ERE motifs [18–23].

Consistent with their broad roles in stress signaling, AP2/ERF-mediated transcriptional regulation is frequently implicated in both biotic and abiotic stress responses [18–26]. For instance, Arabidopsis ERF6 positively contributes to defense against *Botrytis cinerea*, which is associated with reduced hydrogen peroxide (H_2_O_2_) accumulation [27]_._ In contrast, OsERF922 negatively regulates resistance to *Magnaporthe oryzae* while impairing salt stress tolerance [28].

Moreover, numerous *ERF* genes participate in abiotic stress adaptation, including drought and oxidative stress-related pathways [29–33]. ERF74 overexpression upregulates reactive oxygen species (ROS)-detoxifying enzymes and key genes involved in drought response, thereby enhancing drought tolerance [30]. Overexpression of SpERF.B7 increases salt tolerance in both tomato and Arabidopsis [31]. Ectopic expression of pitaya ERF1 in Arabidopsis was found to increase salt tolerance while reducing the accumulation of H_2_O_2_ and O_2_^−^ [32]. Similarly, TERF2/LeERF2 improves freezing tolerance in both tomato and tobacco [34]. In addition, overexpression of *SlERF5* improves tomato tolerance to both drought and salinity [29]. Collectively, these studies demonstrate the important roles of ERF proteins in abiotic stress responses. However, the molecular mechanisms by which ERF proteins regulate drought tolerance remain poorly understood.

Tomato (*Solanum lycopersicum* L.) is a widely cultivated vegetable crop and serves as an important model plant species. Given the high sensitivity of cultivated tomato to water deficit, understanding the molecular mechanisms underlying drought stress tolerance is essential. In our previous work, we identified *SlERF26* as a drought-responsive transcription factor (TF) that showed differential expressed between introgression lines and cultivated tomato under drought conditions [35]. However, the molecular mechanism by which SlERF26 regulates drought tolerance remains largely unclear. In this study, we investigated the functional role of SlERF26 in drought tolerance and delved into its downstream regulatory network in tomato. We further examined whether SlERF26 regulates drought responses through modulating the aquaporin gene *SlPIP2;4*, providing new insights into the transcriptional regulatory mechanisms underlying drought tolerance in tomato.

## Results

### Gene features of ERF26 gene

*SlERF26* (SGN: Solyc06g035700) was initially identified by tomato microarray–based gene expression profiling. The analysis revealed that SlERF26 was differentially expressed in two drought-tolerant *S. pennellii* introgression lines (ILs) compared with the drought-sensitive recurrent parent *S. lycopersicum* cv. M82 under drought stress conditions [35, 36] (Fig. 1a). Compared with well-watered conditions, *SlERF26* expression did not significantly change in M82 following drought treatment but was markedly upregulated in IL2-5 and IL9-1, two drought-tolerant near-isogenic lines [35] (Fig. 1b), implying that *SlERF26* is potentially involved in drought resistance.

**Figure 1:**
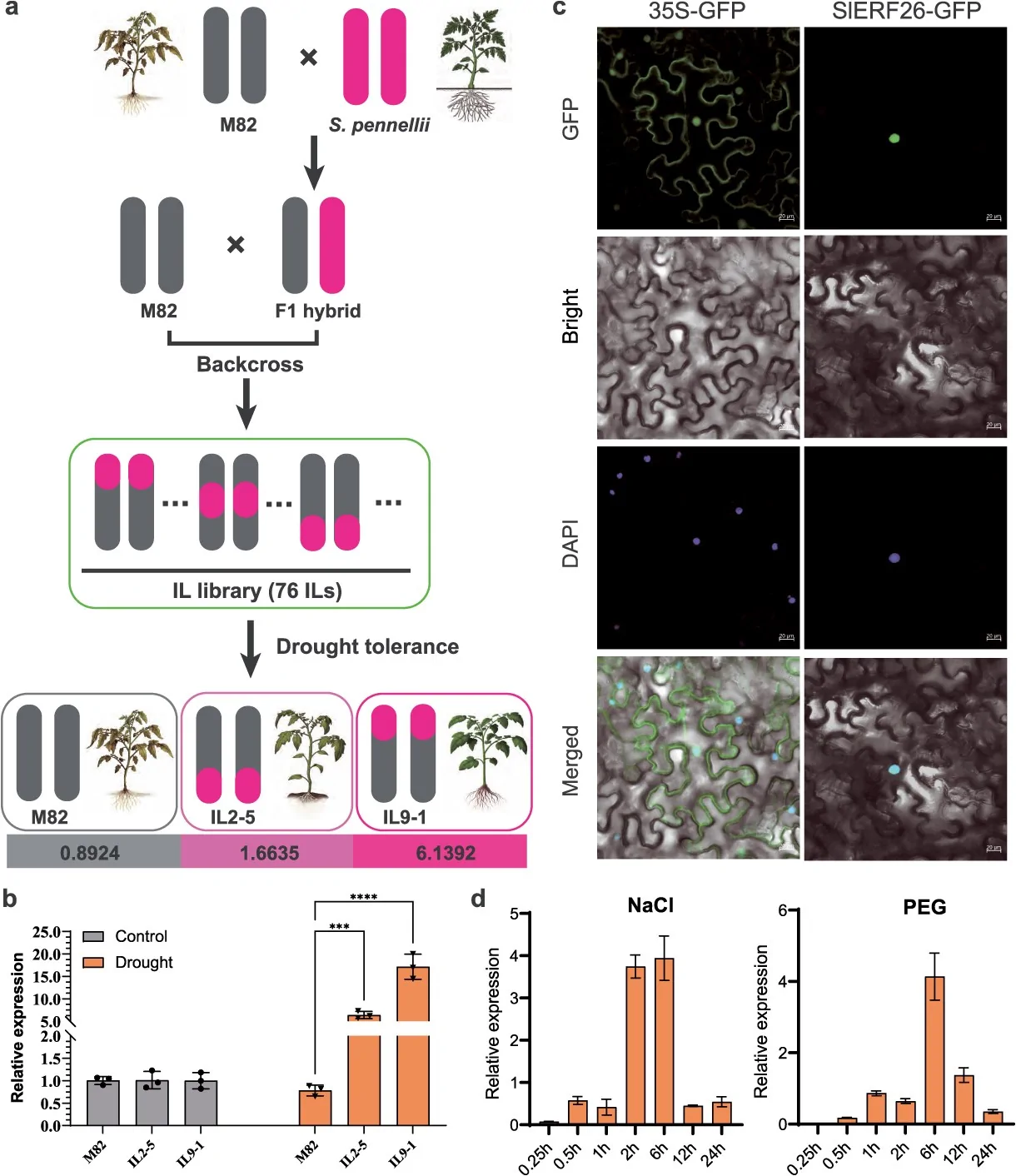
The gene features of *SlERF26*. (a) Schematic overview of the development of the *S. pennellii* introgression line (IL) population. Drought-sensitive *S. lycopersicum* cv. M82 was crossed with drought-tolerant wild tomato *S. pennellii*, and the resulted F1 plants were repeatedly backcrossed to M82 to generate a library of 76 ILs, each carrying defined *S. pennellii* genomic segments [36]. Drought-tolerant ILs were screened, and IL2-5 and IL9-1 were identified as high-performing lines. Microarray-based transcriptome profiling showed that *SlERF26* expression exhibited minimal change in drought-sensitive M82 (fold change 0.8924) but was strongly induced in drought-resistant IL2-5 (1.6635) and IL9-1 (6.1392) under drought stress [35]. (b) RT-qPCR mediated validation of *SlERF26* expression in M82, IL2-5, and IL9-1 under control and drought stress conditions. Drought stress significantly up-regulated *SlERF26* in IL2-5 and IL9-1 but not in M82, consistent with microarray results. Data are presented as mean ± SD from three independent biological replicates. Statistical significance was determined by two-way ANOVA followed by Tukey’s multiple comparison test. Asterisks indicate significant differences compared to M82: ^***^P < 0.001, ^****^P < 0.0001. (c) Subcellular localization of SlERF26 protein into the tobacco epidermal cells. DAPI marks the nucleus, and GFP represents green fluorescent protein; Bright refers to the bright field; Merged indicates the combined images of GFP, DAPI, and the bright field. Scale Bars = 20 μm. (d) RT-qPCR mRNA expression of *SlERF26* in tomato plants under 100 mM NaCl or 100 mM PEG treatment. Transcript levels in stress-treated plants were measured and compared with untreated control plants at the specified time points (in hours). Data represent the means of three biological replicates ± SD.

To explore sequence variation potentially associated with SlERF26 regulation and function, the promoter and coding sequences of *SlERF26* were compared between cultivated tomato (cv. M82) and *S. pennellii*. The promoter and coding region exhibited 92.91% and 94.82% identity, respectively, suggesting that both transcriptional regulation and functional roles in drought response are likely conserved between the two species (Fig. S1). Subsequently, we cloned the gene and found 537 base pairs (bp) coding sequence (CDS) sequence encoding a predicted protein of 178 amino acids. Sequence analysis revealed that SlERF26 harbors a conserved 63-amino acid AP2 domain, including the conserved alanine (A) and aspartic acid (D) residues typical of AP2 family proteins (Fig. S2). Phylogenetic analysis further indicated that SlERF26 clusters closely with StERF26, SvERF26, SsERF26, and CbERF26 (Fig. S3).

To determine the subcellular localization of SlERF26, a SlERF26-GFP fusion protein was transiently expressed in epidermal cells of *Nicotiana benthamiana*. The fluorescence from the SlERF26-GFP was exclusively detected in the nucleus, whereas free GFP was diffusely distributed throughout the cytoplasm and nucleus, indicating that SlERF26 is a nuclear-localized protein (Fig. 1c).

Furthermore, a 3.0-kb sequence upstream of the SlERF26 translation start site was retrieved and analyzed for putative *cis*-regulatory elements using PlantCARE database (Fig. S4). Multiple putative elements associated with plant hormone responsiveness [ABRE, TGA, and trichloroacetic acid (TCA)], light signaling (G-BOX and BOX4), and abiotic stress responses (MBS and LTR) were identified. These predictions suggest that SlERF26 expression may be regulated through the integration of external environmental stimuli and internal hormonal signaling pathways. To test whether *SlERF26* responds to abiotic stress, we analyzed the transcript levels in plants exposed to salt (NaCl) and osmotic (PEG) stresses. Under salt stress, the *SlERF26* transcripts were rapidly induced within 0.5 h and remained elevated for at least 6 h (Fig. 1d). Similarly, PEG treatment led to a rapid upregulation of *SlERF26* transcription in leaves (Fig. 1d). These findings support that *SlERF26* is responsive to both salt and osmotic stress, consistent with the presence of stress-responsive *cis*-elements in its promoter region.

### *SlERF26* overexpression improves drought tolerance in transgenic plants

To investigate the function of *SlERF26* in drought tolerance, four independent homozygous T_2_  *SlERF26*-overexpressing (OE) lines were generated using the CaMV 35S promoter. Reverse transcription quantitative polymerase chain reaction (RT-qPCR) analysis confirmed that *SlERF26* transcript levels in lines OE-18, OE-15, OE-6, and OE-4 were markedly higher than those in the wild type (WT), and these four lines were selected for further experiments (Fig. 2a).

**Figure 2:**
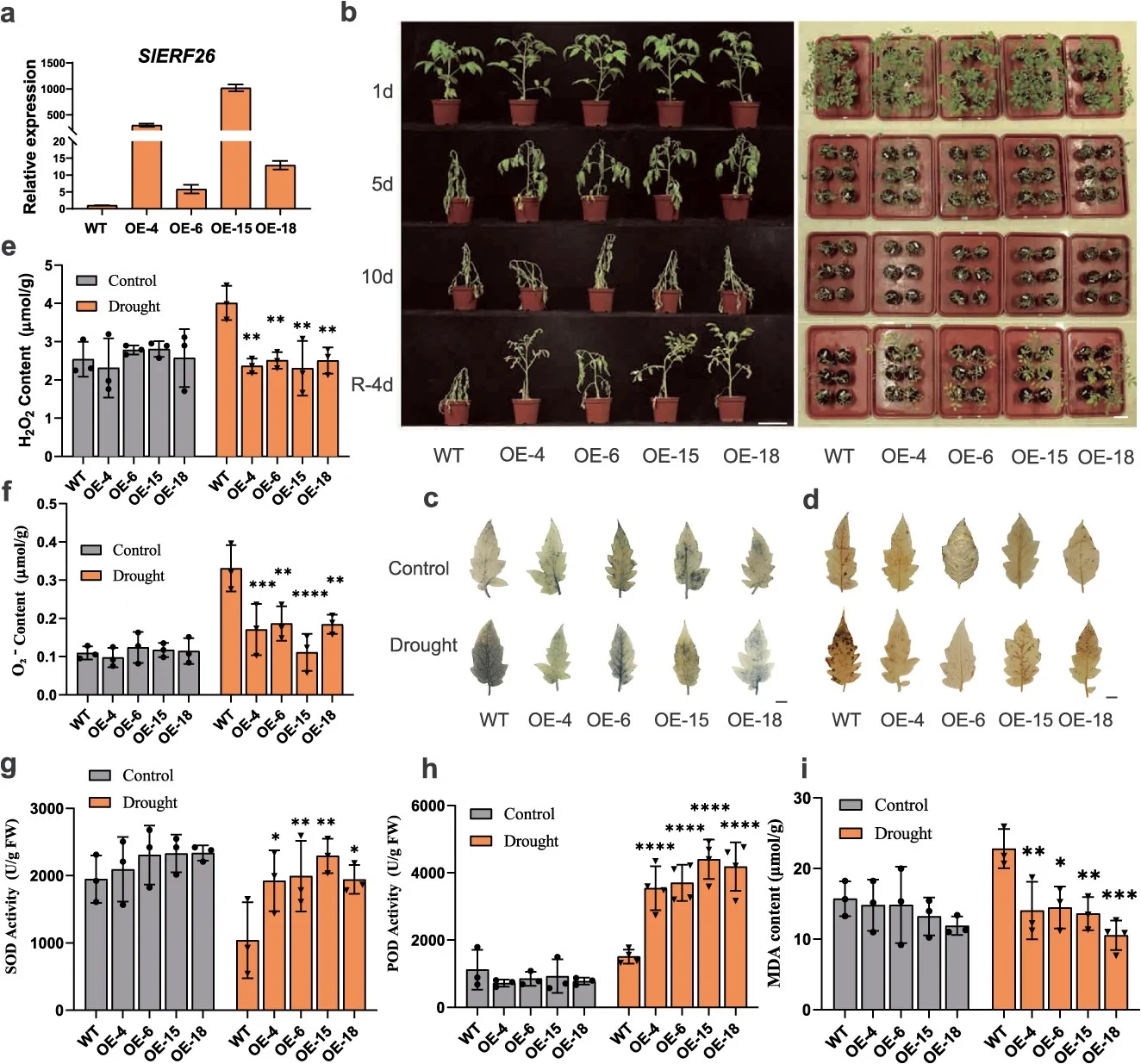
Overexpression of *SlERF26* enhances drought tolerance in tomato. (a) RT-qPCR expression analysis of *SlERF26* in transgenic tomato plants. Transcript levels were quantified in the WT and four independent overexpression lines (OE-4, OE-6, OE-15, and OE-18). Data are presented as mean ± SD from three independent biological replicates. (b) Phenotypic responses of *SlERF26* OE and WT plants under drought conditions and rewatering. Plants were subjected to drought treatment for 1, 5, or 10 d, followed by re-watered the 10-d drought treated plants for subsequent 4 days (R-4d). Representative images are shown. Scale bars = 10 cm. (c and d) Detection of reactive oxygen species in leaves by NBT (c) and DAB (d) staining on the 5 d after drought stress. The second fully expanded leaf of *SlERF26* OE lines and WT were stained with NBT to measure O₂^−^ production (dark-blue formazan deposits) and DAB to measure H₂O₂ accumulation (brown precipitate), under both drought and well-watered conditions. Representative images are shown from three independent biological replicates. Scale bars = 1 cm. (e, f) Quantification of H₂O₂ and O₂^−^ contents in WT and *SlERF26* OE lines under control and drought conditions. (g, h) Activities of SOD and POD in *SlERF26* OE lines and WT plants. (i) Contents of MDA in *SlERF26* OE lines and WT plants. Data are presented as mean ± SD from three independent biological replicates. Statistical significance was determined by two-way ANOVA followed by Tukey’s multiple comparison test. Asterisks indicate significant differences compared to WT: ^*^P < 0.05, ^**^P < 0.01, ^***^P < 0.001, ^****^P < 0.0001.

To evaluate drought tolerance, OE and WT plants were subjected to drought stress by withholding irrigation at the five-leaf stage. At the pre-stress stage (Day 1), no discernible phenotypic differences were observed between the *SlERF26* OE lines and WT plants. After 5 days without watering, WT plants were severely wilted, whereas OE lines only showed mild leaf curling. After 10 days of water deprivation, all plants manifested dehydration symptoms. Nevertheless, following 4 days of re-watering, OE lines recovered significantly, while the WT plants were entirely shriveled (Fig. 2b). To further validate the necessity of SlERF26 for drought resilience, *SlERF26* knockout lines (CR-1, CR-2, and CR-3) generated using CRISPR/Cas9 were subjected to the same drought treatment. Compared with WT plants, the *slerf26* mutant plants showed increased sensitivity to water deficit, with earlier and more severe leaf dehydration and chlorosis (Fig. S5b).

Plants typically generate elevated levels of ROS in response to environmental stress. To assess oxidative stress levels, nitro blue tetrazolium (NBT) and 3,3′-diaminobenzidine (DAB) staining were conducted to visualize O₂^−^ and H₂O₂ accumulation, respectively. Under control conditions, WT and *SlERF26* OE lines showed similar staining intensity. After exposure to drought stress, WT leaves accumulated a substantially larger amount of dark-blue and reddish-brown staining, whereas OE leaves exhibited much weaker staining, which indicated reduced ROS accumulation in the transgenic lines (Fig. 2c and d). Consistently, NBT and DAB staining showed that *slerf26* mutant leaves accumulated higher levels of ROS under drought stress than WT leaves, as evidenced by more intense and widespread precipitates (Fig. S5c and d). Quantitative measurements revealed similar H₂O₂ and O₂^−^ content in WT and *SlERF26* OE lines under control conditions. Under drought stress, H₂O₂ and O₂^−^ levels increased significantly in WT plants, while their accumulation was markedly lower in OE lines (Fig. 2e and f). Although the activities of superoxide dismutase (SOD) and peroxidase (POD) were similar in both genotypes under control condition, both activities were significantly higher in OE lines following drought stress, suggesting enhanced antioxidant capacity in *SlERF26* OE lines (Fig. 2g and h).

Malondialdehyde (MDA), a marker of lipid peroxidation [35], showed no significant difference between the WT and *SlERF26* OE lines under control conditions. After 5 days of drought stress, MDA levels were significantly higher in WT plants than in *SlERF26* OE lines (Fig. 2i). Drought stress increased proline content and electrolyte leakage in both WT and OE plants, but both parameters were notably lower in the OE lines than in WT plants under drought conditions (Fig. 3a and b). Similarly, the chlorophyll content was decreased under drought in both groups of plants; however, these reductions were significantly smaller in the *SlERF26* OE lines compared with WT plants (Fig. 3c). Consistently, *SlERF26* OE lines maintained higher relative water content (RWC) and exhibited significantly lower stomatal conductance (gs) under drought conditions compared with WT (Fig. 3d and e), indicating improved water retention capacity and reduced transpirational water loss. We further quantified stomatal density on the abaxial leaf surface and found that OE lines displayed a significantly lower stomatal density than WT (Fig. 3f and g), which may partially contribute to the reduced gs observed under drought conditions.

**Figure 3:**
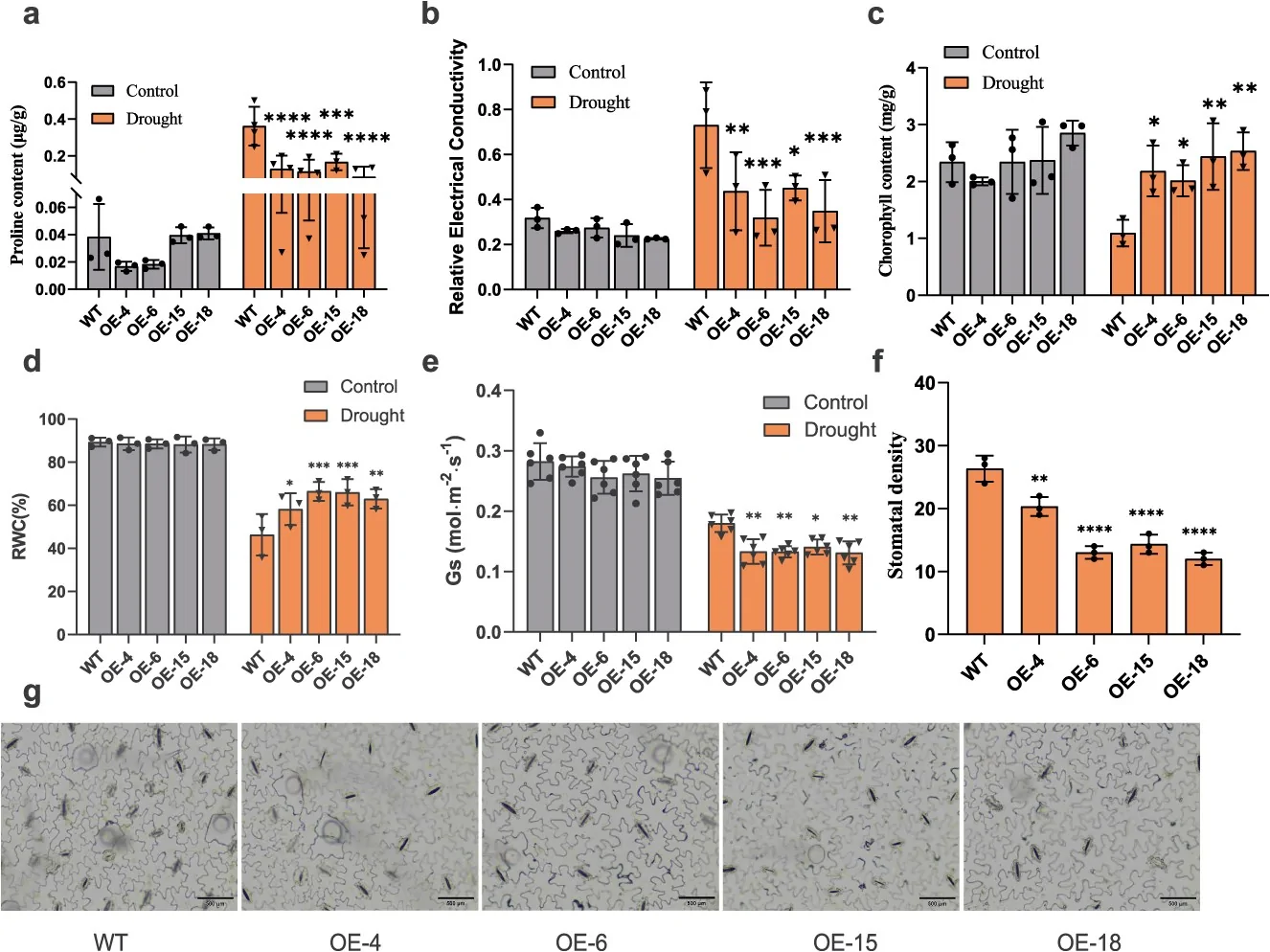
*SlERF26* enhances drought tolerance by regulation of multiple physiological processes. (a–e) Proline content (a), relative electrical conductivity (b), chlorophyll (c), RWC (d), and gs (e) were measured in leaves of *SlERF26* OE lines and WT plants. Samples were collected at 5 days after water withdrawal or under well-watered control conditions. Data are presented as mean ± SD from three independent biological replicates. Statistical significance was determined by two-way ANOVA followed by Tukey’s multiple comparison test. Asterisks indicate significant differences compared to WT: ^*^P < 0.05, ^**^P < 0.01, ^***^P < 0.001, ^****^P < 0.0001. (f, g) Stomatal density analysis (f) and representative images (g) in *SlERF26* OE lines and WT plants under drought stress. Fully expanded leaves were sampled at 5 days after water withdrawal. Data are presented as mean ± SD from three biological replicates. Statistical significance was determined by one-way ANOVA followed by Tukey’s multiple comparison test. Asterisks indicate significant differences compared to WT: ^**^P < 0.01, ^****^P < 0.0001. Scale bars = 500 μm.

Collectively, these results illustrate that *SlERF26* overexpression endows enhanced drought tolerance in tomato by reducing the MDA content, proline accumulation, electrolyte leakage, gs, and stomatal density while maintaining higher chlorophyll content, RWC, and SOD/POD activities, thereby promoting efficient ROS scavenging and improved cellular protection under water deficit.

### *SlERF26* regulates the expression of a series of drought response genes under drought stress

To gain deeper insight of the molecular mechanisms underlying the *SlERF26* mediated improved drought tolerance, RNA sequencing (RNA-Seq) was performed to identify differentially expressed genes (DEGs) between WT and SlERF26 OE lines (OE15 and OE18). A total of 40.41–46.67 million clean reads were obtained for each sample, of which 93.99%–96.14% were successfully mapped to tomato reference genome. The Q30 values ranged from 91.79% to 92.28%, and the average GC content ranged from 41.53% to 42.75% (Tables S1 and S2), indicating that the RNA-seq data were of high quality and suitable for downstream analyses. Correlation analysis and principal component analysis (PCA) demonstrated high reproducibility among biological replicates and clear separation of samples by treatment and genotype (Fig. S6), supporting the reliability of subsequent differential expression analysis.

Global transcriptome profiling revealed distinct transcriptional differences between drought treatment (DT) and control (CK) conditions, as well as between the WT and OE plants. This indicates that overexpression of the *SlERF26* alone can modify the expression levels of numerous downstream genes (Fig. 4a). Under drought stress, 326 DEGs were identified in OE-15 compared to WT, including 96 upregulated and 230 downregulated genes, whereas 124 DEGs (27 upregulated and 97 downregulated) were identified in OE-18 (Fig. S7). In addition, comparison of DT versus CK within each OE line revealed 2162 DEGs shared by OE-15 and OE-18, representing a core set of drought-responsive genes consistently affected in the *SlERF26* OE background (Fig. 4b). Kyoto Encyclopedia of Genes and Genomes (KEGG) enrichment analysis demonstrated these DEGs were mainly involved in carbon metabolism, glutathione metabolism, mitogen-activated protein kinase (MAPK) signaling, and peroxisome function (Fig. 4c), suggesting that SlERF26 participates in multiple stress-responsive pathways.

**Figure 4:**
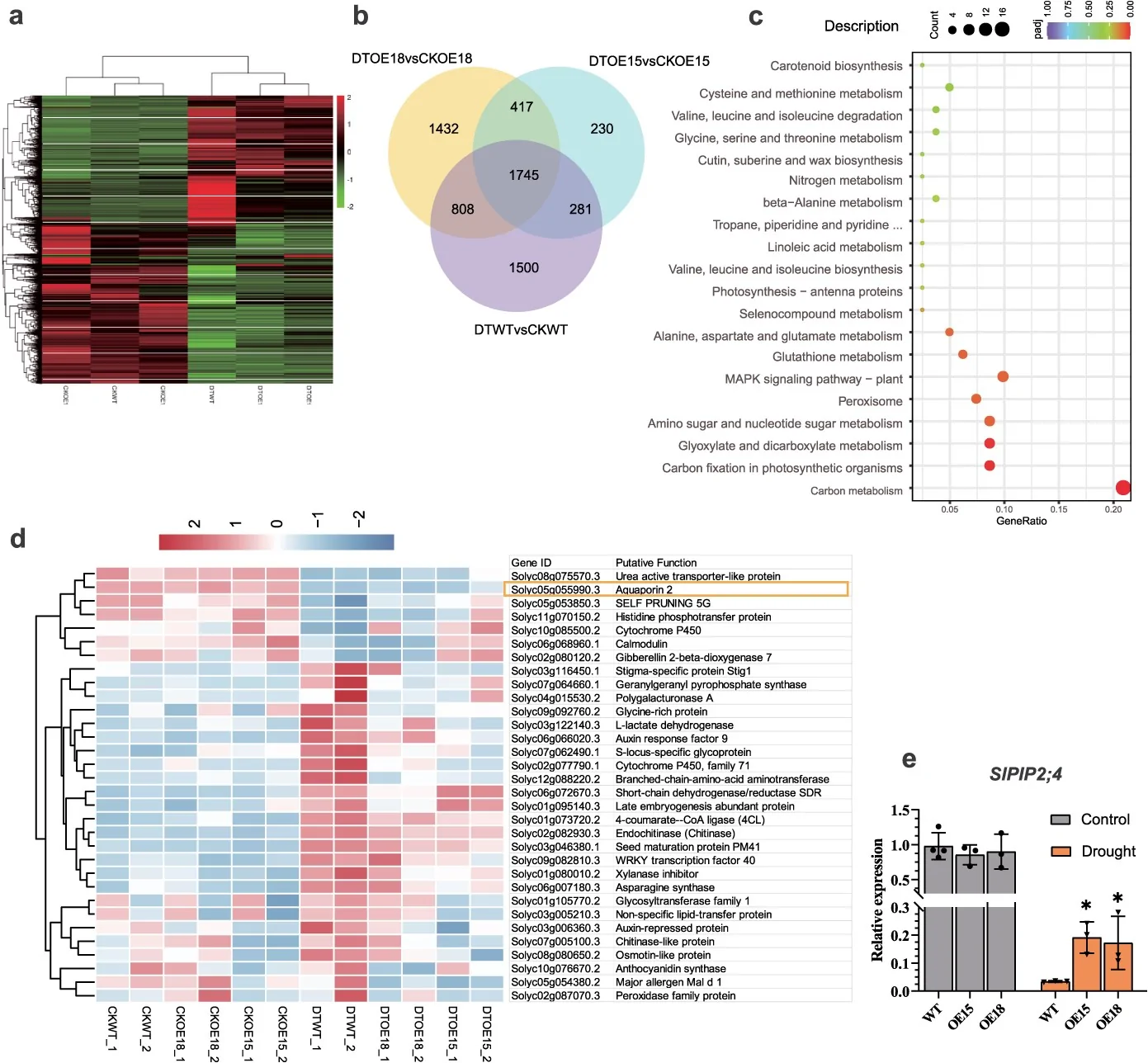
Transcriptome analysis of DEGs in *SlERF26* transgenic plants under drought stress. (a) Heatmap showing hierarchical clustering of DEGs in SlERF26 OE lines (OE-15, OE-18) and WT plants under drought stress. Each cell represents the normalized relative expression level of a DEG, with lower and higher expression values indicated according to the scale bar. (b) Venn diagram showing shared and unique DEGs between the three DT (Drought) vs. CK (Control) comparisons (DTOE15 vs. CKOE15, DTOE18 vs. CKOE18, and DTWT vs. CKWT). (c) KEGG pathway enrichment analysis for DEGs. The *x*-axis shows the percentage of DEGs mapped to each pathway, while the *y*-axis enumerates the significantly enriched KEGG pathways. The gene count denotes the number of DEGs within each pathway. The adjusted *p*-value indicates the enrichment significance, with lower adjusted *p*-values representing greater significance. (d) Clustering analysis was performed on DEGs whose promoters contained the DRE or GCC elements. Rows were clustered using hierarchical clustering with Euclidean distance and complete linkage. (e) The expression level of *SlPIP2;4* in *SlERF26* OE-15, OE-18, and WT were measured after 5 days of drought treatment or well-watered control. Data are presented as mean ± SD from three independent biological replicates. Statistical significance was determined by two-way ANOVA followed by Tukey’s multiple comparison test. Asterisks indicate significant differences compared to WT: ^*^P < 0.05.

Promoter analysis of the DEGs identified 32 genes containing DRE or GCC *cis*-elements, suggesting that these genes may be transcriptionally regulated by SlERF26 (Fig. 4d). To clarify the potential regulatory pathways associated with SlERF26-mediated drought tolerance, we focused on identifying functional genes that could directly involve in plant protection and drought stress mitigation. Among them, *SlPIP2;4* was downregulated by drought stress in WT plants while upregulated in *SlERF26* OE lines under the same conditions (Fig. 4e). Given that aquaporin genes play important roles in drought tolerance and antioxidation in plants [37], *SlPIP2;4* was selected for further investigation. In addition to *SlPIP2;4*, a POD family gene containing a DRE motif in its promoter was also identified from the RNA-seq dataset (Fig. 4d). RT-qPCR analysis confirmed the differential expression of this *POD* gene in *SlERF26* OE lines, suggesting that it may function as a downstream target of SlERF26 (Fig. S12c). These results indicate that SlERF26 may regulate ROS homeostasis through transcriptional regulation of ROS-related genes*.* Furthermore, RT-qPCR validation of 12 representative genes was consistent with the RNA-seq results, which confirmed the reliability of the transcriptomic data (Fig. S8).

### SlERF26 directly binds to the *SlPIP2;4* promoter

We analyzed the promoter region of *SlPIP2;4* and identified a DRE motif located ~741 bp upstream of the transcription start site. Dual-luciferase assays showed that SlERF26 significantly activated a 2000-bp promoter reporter (SlPIP2;4pro-2 K: LUC). Consistently, SlERF26 also enhanced the activity of a 161-bp fragment containing DRE motif (SlPIP2;4pro-DRE: LUC), whereas mutation of the DRE motif markedly attenuated this activation, indicating that the DRE element is required for SlERF26-mediated transactivation (Fig. 5a–c). To directly assess binding, we carried out a Y1H assay. SlERF26 specifically interacted with the DRE-containing promoter fragment, while no interaction was detected when the DRE motif was mutated (Fig. 5d). Moreover, EMSA confirmed that the purified HIS-SlERF26 protein directly bound to DNA probes containing the 5′-TGTCGG-3′ DRE motif, but not to probes with the mutated sequence (Fig. 5e). Collectively, these findings demonstrate that SlERF26 directly targets the DRE motif in the *SlPIP2;4* promoter to regulate its transcription.

**Figure 5:**
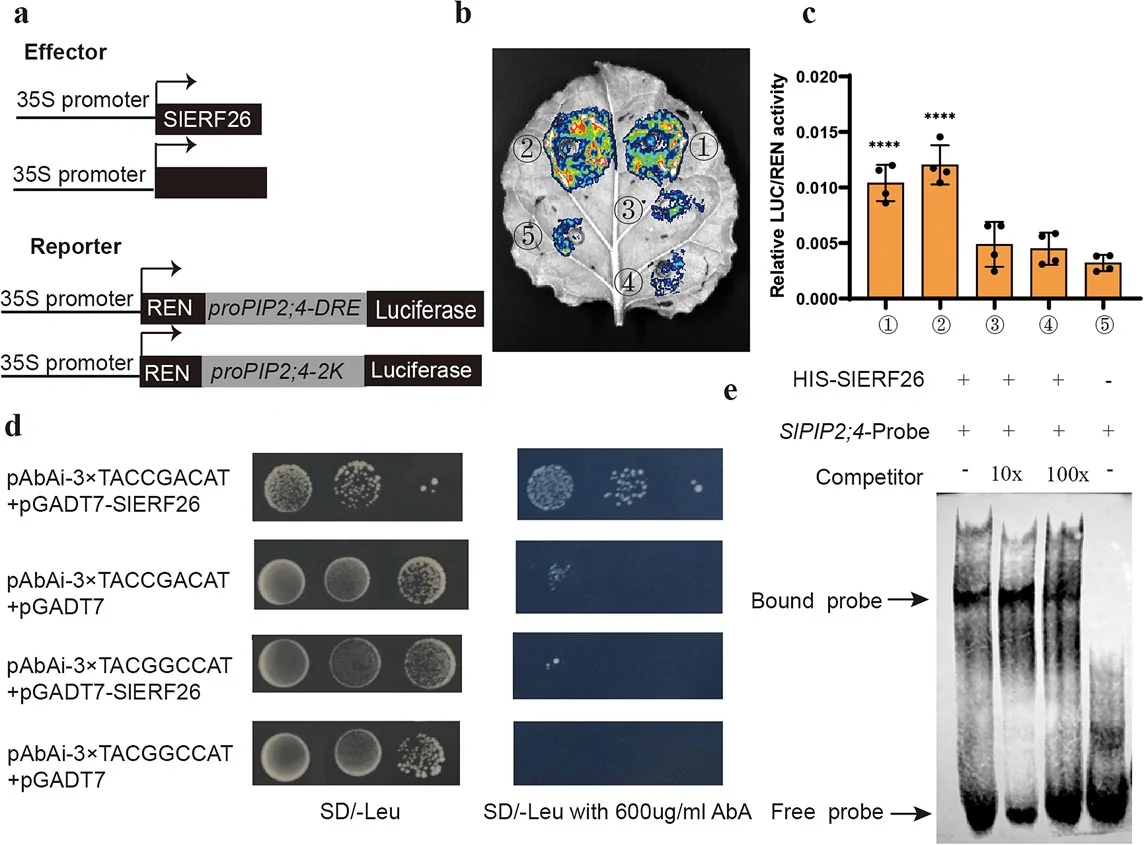
SlERF26 directly binds to the promoter of *SlPIP2;4* and regulates its expression. (a) A 161-bp fragment of the *SlPIP2;4* promoter containing the DRE motif (proPIP2;4-DRE) and a 2000-bp upstream promoter region (proPIP2;4-2K) were separately fused to the firefly luciferase (LUC) gene to generate reporter constructs. The SlERF26 coding sequence was cloned from the pGADT7-Rec2 vector into the effector construct under the control of a constitutive promoter. (b) Dual-luciferase reporter assays to assess the transactivation of the *SlPIP2;4* promoter by SlERF26 in *N. benthamiana* leaves. Reporter constructs containing the DRE fragment (proPIP2;4-DRE), mutated DRE fragment (proPIP2;4-DRE-mut), or 2000-bp upstream promoter (proPIP2;4-2 K) were co-expressed with the effector construct (62-SK-ERF26) or empty vector control (62-SK). The combinations were as follows: ① 62-SK-ERF26 + proPIP2;4-DRE; ② 62-SK-ERF26 + proPIP2;4-2 K; ③ 62-SK-ERF26 + proPIP2;4-DRE-mut; ④ 62-SK + proPIP2;4-DRE; ⑤ 62-SK + proPIP2;4-2 K. (c) Relative LUC/REN activity was further quantified in tomato protoplasts. The data are presented as the mean ± standard error (*n* = 4). Asterisks denote significant differences when compared to the control (⑤): ^****^P < 0.0001. Statistical analysis was carried out using Student’s *t*-test. (d) A Y1H assay was conducted to demonstrate SlERF26 binding to DRE motifs. Three tandem repeats of the DRE motif or its mutant were cloned into the pAbAi and co-transformed with pGADT7–Rec2– SlERF26 into Y1HGold. The transformants were grown to OD600 of 0.2, serially diluted (1, 10^−1^, 10^−2^), and spotted onto SD/-Leu medium containing 600 ng/ml aureobasidin A for selection. (e) EMSA was performed using a biotin-labeled probe containing the DRE motif (TGTCGG) from the *SlPIP2;4* promoter and recombinant 6×His-tagged SlERF26 protein. Binding specificity was confirmed through competition with an unlabeled probe (competitor) and by testing specificity with a labeled mutant probe. Three biological replicates produced similar results.

### *SlPIP2;4* positively regulates drought tolerance in tomato

*SlPIP2;4* was silenced using a virus-induced gene silencing (VIGS) system (pTRV—*SlPIP2;4*) as a preliminary assay of its role in drought tolerance. After 7 days of drought stress, *SlPIP2;4* silenced plants exhibited higher sensitivity to water deficit compared with pTRV control plants. Consistently, silenced plants also displayed stronger NBT and DAB staining, indicating increased accumulation of ROS (Fig. S9a–d). To corroborate these findings, two independent CRISPR/Cas9 knockout lines of *slpip2;4* were generated in tomato, with 1-bp and 3-bp deletions respectively. The 1-bp deletion caused a frameshift leading to a premature stop codon, whereas the 3-bp deletion resulted in loss of Leu79 without changing the reading frame (Fig. 6a). To assess the potential consequence of the loss of Leu79, we performed sequence and structural analyses. Multiple sequence alignment revealed that the C–G–X–V–G–L–L region is highly conserved among plant PIP aquaporins (Fig. S10d). Transmembrane topology prediction using TMHMM indicated that Leu79 is located near the N-terminal region of Loop A, close to the second transmembrane helix. Consistent with this, structural comparison based on AlphaFold modeling suggested local conformational changes in Loop A and adjacent regions in the mutant protein, suggesting a potential influence on channel architecture (Fig. S10e and f).

**Figure 6:**
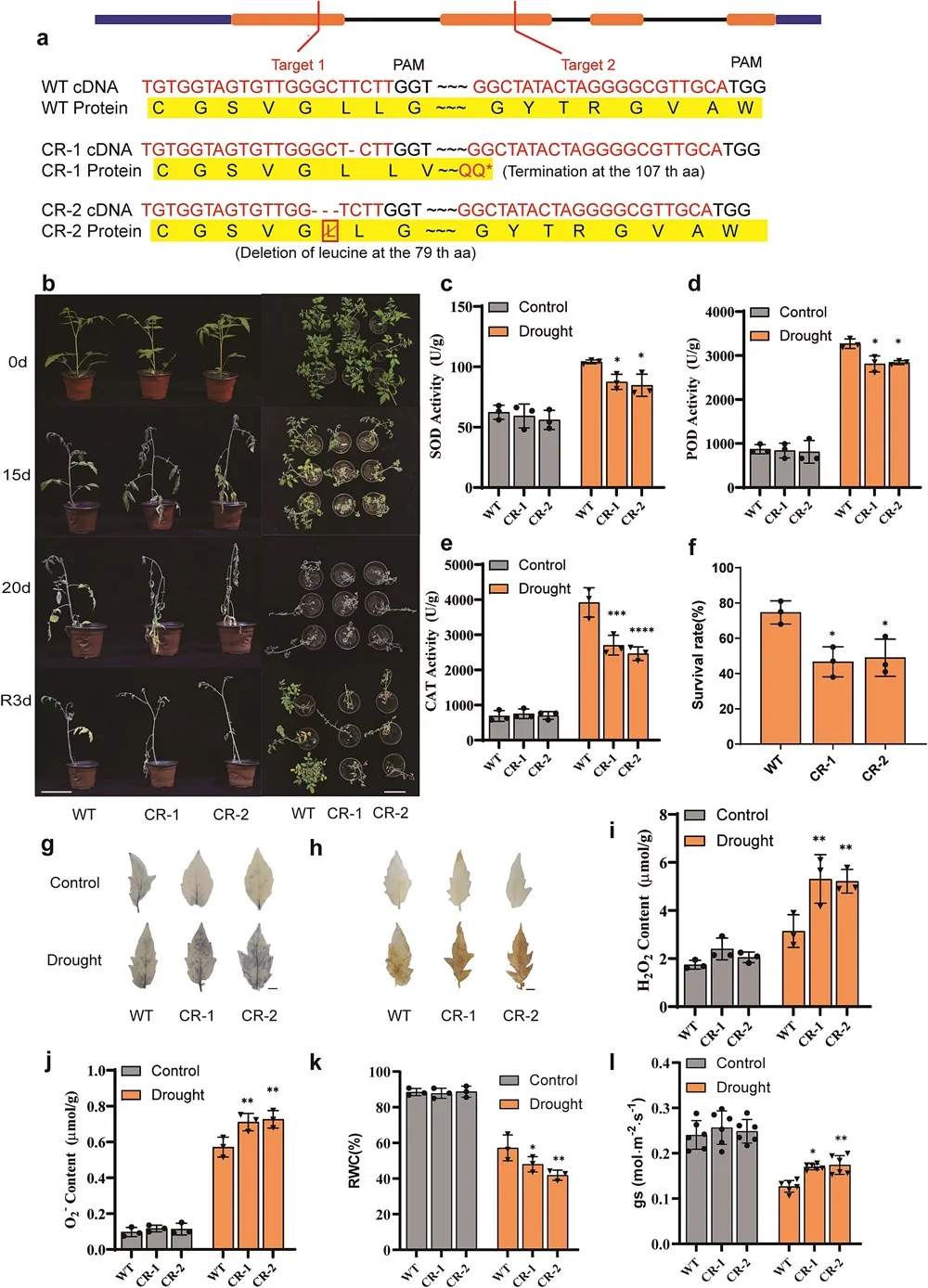
SlPIP2;4 positively regulates drought tolerance in tomato. The gene structure of *SlPIP2;4* and the genomic sequences of *slpip2;4* mutant plants (CR-1 and CR-2) are presented. Knockout lines with frameshift mutations in the target region were confirmed by genomic sequencing. The dashed line (-) represents a 1-bp deletion in the CR-1 line and a 3-bp deletion in the CR-2 line, respectively. The corresponding amino acid sequences are shown below each cDNA sequence, with deleted residues indicated by boxes. The asterisk (*) indicates a premature stop codon. Seedling phenotypes of *slpip2;4* mutant plants (CR-1 and CR-2) were evaluated under drought stress, with WT serving as the control. Scale bars =10 cm. (c–e) Activities of antioxidant enzymes, including SOD (c), POD (d), and CAT (e), were measured in WT and *slpip2;4* mutant plants under control conditions (gray bars) and after drought treatment (orange bars). Data are presented as mean ± SD from three independent biological replicates. Statistical significance was determined by two-way ANOVA followed by Tukey’s multiple comparison test. Asterisks indicate significant differences compared to WT: ^*^P < 0.05, ^**^P < 0.01, ^***^P < 0.001, ^****^P < 0.0001. (f) Survival rates of WT and *slpip2;4* mutant plants after recovery from drought. Data are presented as mean ± SD from three biological replicates. Statistical significance was determined by one-way ANOVA followed by Tukey’s multiple comparison test. Asterisks indicate significant differences compared to WT: ^*^P < 0.05. (g, h) Tissue staining of WT and *slpip2;4* mutant plants under drought stress was conducted using NBT (g) and DAB (h). Drought tolerance assays were independently repeated three times, yielding consistent results, and a representative image is presented here. Scale bars= 1 cm. (i–l) Changes in H₂O₂ (i) and O_2_^−^ (j) contents, RWC (k), and gs (l) were measured in tomato seedlings of different *slpip2;4* mutant plants under control conditions (gray bars) and drought treatment (orange bars). Data are presented as mean ± SD from three independent biological replicates. Statistical significance was determined by two-way ANOVA followed by Tukey’s multiple comparison test. Asterisks indicate significant differences compared to WT: ^*^P < 0.05, ^**^P < 0.01, ^***^P < 0.001, ^****^P < 0.0001.

Phenotypic analysis showed that after 15 days of drought stress, *slpip2;4* mutant plants displayed increased sensitivity to drought stress than WT plants (Fig. 6b). The activities of antioxidant enzymes (SOD, POD, and catalase (CAT)) were comparable between WT and *slpip2;4* mutant plants under control conditions. However, under drought stress, activities of all three enzymes were significantly reduced in *slpip2;4* mutant plants relative to WT (Fig. 6c–e). After 25 days of drought stress and subsequent rehydration, survival rates were markedly lower in *slpip2;4* mutant plants (46% for CR-1 and 49% for CR-2) than in WT plants (Fig. 6f). Histochemical staining further showed stronger dark-blue (NBT) and brown (DAB) precipitates in leaves of *slpip2;4* mutant plants than in WT plants under drought conditions, suggesting increased ROS accumulation (Fig. 6g and h). Quantitative measurements confirmed that H₂O₂ and O₂^−^ levels were similar between genotypes under control conditions but were significantly higher in *slpip2;4* mutant plants after drought treatment (Fig. 6i and j). Although *slpip2;4* mutant plants exhibited smaller stomatal apertures under drought conditions (Fig. S11), they showed lower RWC and higher gs than WT plants (Fig. 6k and l), indicating impaired water status regulation and altered gas-exchange capacity.

### SlERF26 acts upstream of *SlPIP2;4* to regulate drought tolerance

To investigate the genetic relationship between *SlERF26* and *SlPIP2;4* in drought tolerance, hybrid plants were generated by crossing *SlERF26* OE lines with *slpip2;4* mutant plants (OE6 × CR1 and OE18 × CR2). Subsequently, the expression patterns of *SlERF26* and *SlPIP2;4* in these hybrid plants were subsequently examined by RT-qPCR. *SlERF26* transcript levels were significantly higher in OE6/CR1 and OE18/CR2 plants, whereas *SlPIP2;4* expression was reduced relative to WT plants (Fig. 7a and b). The resulting plants were then exposed to drought stress. After 18 and 23 days of drought treatment, OE6/CR1 and OE18/CR2 plants displayed reduced drought tolerance compared with *SlERF26* OE lines but still showed higher tolerance than WT and *slpip2;4* mutant plants (CR1 and CR2) (Fig. 7c). Consistently, OE6/CR1 and OE18/CR2 exhibited intermediate survival rates (Fig. S12a), which were lower than those of *SlERF26* OE lines but higher than those of WT and *slpip2;4* mutant plants. Consistent with these phenotypic differences, RWC showed a similar trend. *SlERF26* OE lines maintained the highest water status, whereas *slpip2;4* mutant plants exhibited the most severe dehydration under drought stress (Fig. S12b). These intermediate phenotypes suggest that the contribution of SlERF26 to drought tolerance is at least partially dependent on SlPIP2;4.

**Figure 7:**
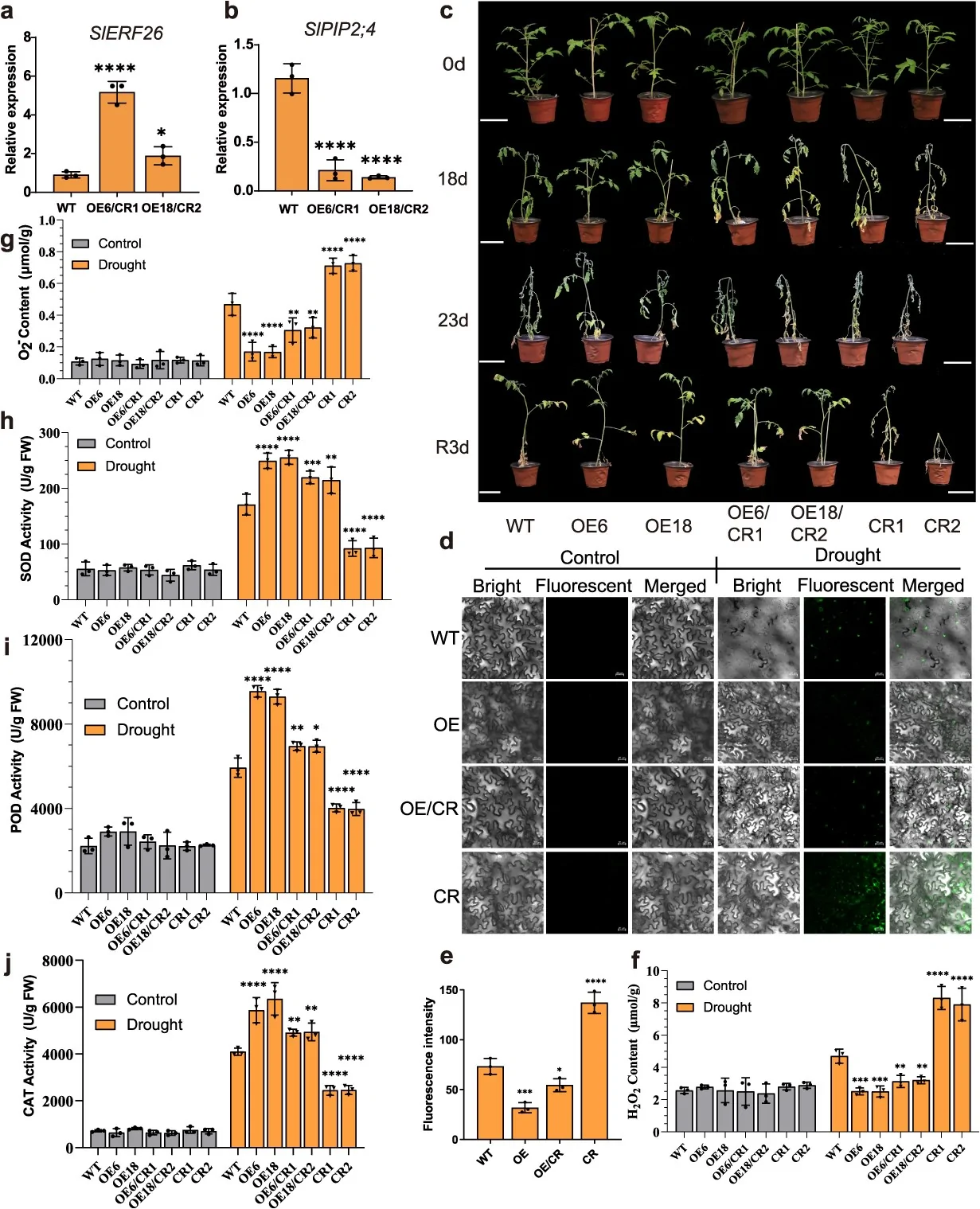
*SlPIP2;4* acts downstream of *SlERF26* to regulate drought tolerance in tomato. (a and b) The relative transcript levels of *SlERF26* (a) and *SlPIP2;4* (b) were quantified by RT-qPCR in hybrid plants (OE6/CR1 and OE18/CR2) compared with WT after 18 days of well-watered growth. Data are presented as mean ± SD from three biological replicates. Statistical significance was determined by one-way ANOVA followed by Tukey’s multiple comparison test. Asterisks indicate significant differences compared to wild type (WT): ^*^P < 0.05, ^****^P < 0.0001. (c) Phenotypes of *SlERF26* (OE6, OE18), *slpip2;4* (CR1, CR2), and hybrid plants (OE6/CR1, OE18/CR2) were observed. These plants were grown in soil, exposed to drought stress for 0–23 days, and then rehydrated for 3 days (R3d) after 23 days of drought stress. Scale bars = 10 cm. (d) Representative fluorescence images of H₂DCFDA signals in leaves. Scale bars= 20 μm. (e) Quantification of H₂DCFDA fluorescence intensity under drought stress in different lines. Data are presented as mean ± SD from three biological replicates. Statistical significance was determined by one-way ANOVA followed by Tukey’s multiple comparison test. Asterisks indicate significant differences compared to WT: ^*^P < 0.05, ^***^P < 0.001, ^****^P < 0.0001. (f–j) H_2_O_2_ (f) and O_2_^−^ (g) content, SOD (h), POD (i), and CAT (j) activities were determined in tomato seedlings of different lines under control and drought condition. Data are presented as mean ± SD from three independent biological replicates. Statistical significance was determined by two-way ANOVA followed by Tukey’s multiple comparison test. Asterisks indicate significant differences compared to WT: ^*^P < 0.05, ^**^P < 0.01, ^***^P < 0.001, ^****^P < 0.0001.

To elucidate the physiological basis underlying these phenotypic differences, ROS accumulation was assessed using multiple complementary approaches. Under drought conditions, *SlERF26* OE lines accumulated significantly lower levels of ROS, whereas the highest ROS accumulation was detected in *slpip2;4* mutant plants, with hybrid plants and WT displaying intermediate levels, as shown by H₂DCFDA fluorescence analysis (Fig. 7d and e). Consistently, H₂O₂ and O₂^−^ contents were elevated in OE6/CR1, OE18/CR2, and WT plants than in *SlERF26* OE lines but remained lower than those in *slpip2;4* mutant plants (Fig. 7f and g). These patterns were further supported by NBT and DAB staining (Fig. S12e and f).

Consistent with the ROS accumulation patterns, the transcript levels of genes encoding antioxidant enzymes (*SOD* and *POD*) were significantly upregulated in *SlERF26* OE plants but progressively declined in hybrid lines, WT, and *slpip2;4* mutant plants (Fig. S12c and d). Correspondingly, OE6/CR1 and OE18/CR2 exhibited intermediate antioxidant enzyme activities, including SOD (Fig. 7h), POD (Fig. 7i), and CAT (Fig. 7j), which were higher than those of WT and *slpip2;4* mutant plants but lower than those of *SlERF26* OE lines.

Collectively, these findings support a model in which SlERF26 acts upstream of *SlPIP2;4* to enhance drought tolerance, likely by coordinating ROS homeostasis, antioxidant capacity, and water retention to mitigate oxidative damage under drought stress.

## Discussion

ERF TFs belong to a large and highly versatile family of plant transcriptional regulators that mediate various responses to developmental cues and environmental stresses. In tomato, 85 *ERF* genes have been identified, and a phylogenetic analysis has been performed together with their *Arabidopsis thaliana* orthologs [16]. *SlERF26* was originally identified from drought-tolerant *S. pennellii* introgression lines and exhibited markedly stronger drought-induced expression in IL2-5 and IL9-1 than in M82 [35] (Fig. 1a). Sequence and subcellular localization analyses verified that SlERF26 encodes a nucleus-localized AP2/ERF TF possessing a conserved 63-amino-acid AP2 domain (Figs 1c and S1b). The evolutionary conservation of ERF26 orthologs across various species suggests that SlERF26 may execute conserved regulatory functions in plant adaptation to abiotic stresses (Fig. S1c), akin to other ERFs that govern stress responses in Arabidopsis, maize, and soybean [25, 38, 39]. Expression profiling in our study further revealed that *SlERF26* was strongly induced by drought and salt stress, underscoring its participation in abiotic stress signaling (Fig. 1d). This inducible expression pattern is consistent with previous reports indicating that AP2/ERF TFs integrate hormonal signals (e.g. abscisic acid (ABA,) ethylene) and environmental cues to coordinate adaptive responses [23, 30, 33, 40, 41]. Together, these observations suggest that SlERF26 is a functionally relevant ERF regulator in tomato with the potential to regulate downstream stress-responsive gene networks.

### *SlERF26* enhances drought tolerance in tomato

Drought stress is among the most devastating environmental factors that negatively impact agricultural production [42]. Plants have developed multiple defense strategies, such as morphological adaptations, osmotic adjustment, maintenance of membrane integrity, and enhancement of ROS-scavenging capacity. Extensive studies have demonstrated that ERF TFs are involved in regulating ROS signaling and proline metabolism [22, 23, 25]. In tomato, Overexpression of *LeERF1* and *LeERF2* increases proline accumulation and reduces the levels of MDA, an indicator of lipid peroxidation [43]. Consistent with this, *SlERF26* OE lines accumulated lower levels of MDA, H_2_O_2_, and O_2_^−^ under drought stress while maintaining higher chlorophyll content and enhanced activities of antioxidant enzymes, including SOD and POD (Figs 2 and 3). These findings suggest that *SlERF26* strengthens cellular antioxidant defense system, thereby alleviating oxidative damage and maintaining photosynthetic efficiency under water-deficit conditions. Furthermore, the transcript levels of genes encoding antioxidant enzymes, including *POD* and *SOD*, were significantly upregulated in *SlERF26* OE lines under drought stress, consistent with the increased enzymatic activities. This suggests that SlERF26 may enhance antioxidant capacity at both the transcriptional and enzymatic levels, contributing to more efficient ROS detoxification. In addition to enhanced antioxidant capacity, *SlERF26* OE lines maintained significantly higher RWC and exhibited reduced gs under drought stress, indicating improved water retention and decreased transpirational water loss. These physiological traits are likely associated with the enhanced ROS homeostasis observed in OE lines, as reduced oxidative damage may help sustain membrane integrity and cellular water balance during dehydration stress. Interestingly, *SlERF26* OE lines also displayed reduced stomatal density (Fig. 3f), suggesting a potential role in stomatal development. A lower stomatal density may contribute to reduced gs and improved water conservation. However, because stomatal density is largely determined during leaf development, this difference likely represents a developmental trait rather than an immediate drought-induced response. Therefore, while altered stomatal density may partially contribute to drought tolerance, it is unlikely to fully account for the observed physiological and stress-resistance phenotypes.

Notably, *SlERF36*, another ERF TF containing an EAR motif, has been reported to negatively regulate stomatal density in tomato [44]. This indicates that modulation of stomatal development may represent a conserved function in ERF TFs.

Collectively, our results indicate that SlERF26 enhances drought tolerance through coordinated regulation of ROS homeostasis, antioxidant capacity at both transcriptional and enzymatic levels, and water retention, thereby improving plant resilience under water-deficit conditions.

### Transcriptomic profiling reveals *SlERF26*-regulated pathways under drought stress

To explore the molecular mechanisms underlying *SlERF26*-mediated drought tolerance, we performed comparative transcriptomic analysis was conducted between OE and WT plants under drought conditions. Two thousand one hundred sixty-two DEGs were identified between the plants under drought conditions and the control group due to *SlERF26* overexpression (Fig. 4b). KEGG pathway analysis suggested that these DEGs were mainly associated with carbon metabolism, MAPK signaling, and peroxisome-related pathways (Fig. 4c), highlighting broad transcriptional reprogramming in OE plants during drought stress. Carbon metabolism is essential for plant growth, development, and yield formation. It involves key processes including photosynthetic CO_2_ assimilation, carbohydrate biosynthesis, catabolism, and interconversion [45]. Drought stress typically induces oxidative damage, triggers stomatal closure, and restricts shoot growth [46]. *ERF110* enhances cold tolerance in citrus by modulating sugar and sterol accumulation [26]. The *SlERF26* OE lines exhibited higher chlorophyll content and a reduced stomatal density, which may help preserve photosynthetic capacity and improve water conservation under drought stress. Therefore, *SlERF26* might precisely adjust carbon allocation to maintain cellular homeostasis, thereby improving drought tolerance. The MAPK signaling pathway plays a central role in plant responses to drought stress, actively engaging in stomatal regulation, ROS metabolism, and the activation of stress-responsive TFs [1, 2]. In both tomato and Arabidopsis, multiple MAPKs are promptly activated under drought or osmotic stress. Subsequently, they also regulate downstream TFs and stress-inducible genes to enhance drought tolerance [47, 48]. To gain further insight into the downstream regulatory network associated with SlERF26, we analyzed the expression patterns of 32 selected genes and performed functional annotation based on the Solanaceae genome database. Functional categorization revealed that these genes could be broadly grouped into four major classes: membrane transport, hormone signaling, ROS scavenging/defense, and primary/secondary metabolism. Notably, genes involved in membrane transport included an aquaporin and a solute transporter, which are potentially associated with transmembrane movement of water and small molecules movement. Given previous evidence that certain aquaporins facilitate H₂O₂ diffusion across membranes [49, 50], this observation supports a mechanistic connection between SlERF26-mediated transcriptional regulation and intracellular ROS distribution.

In addition, several genes related to hormone signaling pathways were identified, including gibberellin 2-oxidase, auxin response factor 9, and a histidine phosphotransfer protein. The presence of these genes suggests that SlERF26 may participate in crosstalk between stress responses and phytohormone signaling, potentially modulating plant growth and adaptation under stress conditions.

Importantly, a substantial proportion of the selected genes were associated with ROS scavenging and stress defense, such as peroxidases, chitinases, and osmotin-like proteins. These proteins are well known to function in ROS detoxification and enhancing stress tolerance, indicating that the SlERF26 regulatory network may contribute to maintaining ROS homeostasis [51–55]. This is consistent with the physiological phenotypes observed in *SlERF26* OE plants. Taken together, these results suggest that SlERF26 functions as an upstream regulator coordinating multiple biological processes, including membrane transport, hormone signaling, ROS detoxification, and metabolic reprogramming. Rather than acting through a single downstream target, SlERF26 likely modulates a broader transcriptional network, with SlPIP2;4 serving as a key node linking H₂O₂ transport to ROS homeostasis. This integrated regulatory framework provides a mechanistic basis for understanding how SlERF26 enhances stress tolerance in tomato.

### SlERF26 directly regulates *SlPIP2;4* to modulate redox homeostasis

ERF proteins are TFs that regulate stress-responsive genes by binding to their promoters, thereby enhancing stress tolerance. The GCC-box (AGCCGCC) is a well-characterized *cis*-element that is bound by several ERF proteins, whereas others ERFs preferentially bind the DRE (A/GCCGAC). Some ERFs display dual DNA-binding specificity and are capable of interacting with both motifs. For example, ERF1 was demonstrated to specifically bind either the GCC-box or DRE/CRT elements in a stress-dependent manner, facilitating responses to distinct abiotic stresses [38]. In tobacco, overexpression of *NtERF172* increases drought tolerance by controlling *NtCAT* expression, potentially through DRE element binding in the promoter region [40]. Therefore, identifying the downstream targets of ERFs in tomato will offer crucial insights into their functional roles and the molecular mechanisms underlying drought tolerance regulation. In this study, we identified SlPIP2;4, a plasma membrane aquaporin, as a key downstream component of the SlERF26 regulatory network (Fig. 4d and e). Dual-luciferase reporter assays and EMSA verified that SlERF26 directly binds to the DRE motifs in the *SlPIP2;4* promoter, thereby transcriptionally activating this gene (Fig. 5).

Aquaporins (AQPs) are major membrane proteins mediating transport of water and various small neutral solutes across plant cellular membranes. Phylogenetic and sequence analyses further divide PIPs into two subfamilies, PIP1 and PIP2. PIP2 aquaporins are generally regarded as having higher intrinsic water channel activity than PIP1 isoforms [56]. ROS can induce cellular damage by promoting lipid peroxidation, nucleic acid oxidation, and the loss of enzymatic activity [57]. Increasing evidence suggests that certain aquaporins act as peroxiporins, facilitating transmembrane transport of H₂O₂, thus modulating redox signaling and influencing the activity of downstream enzymes [6, 58–60]. The kinase OST1 phosphorylates Ser121 of aquaporin PIP2;1, a post-translational modification participates in ABA-and ROS-mediated stomatal closure [59]. In addition to redox signaling, AQPs are crucial for maintaining cellular water homeostasis under abiotic stress [61, 62]. *SlPIP2;1*, and *SlPIP2;5* have been reported to improve drought tolerance by achieving higher hydraulic conductivity levels [63]. Overexpression of *SiPIP2;4* promotes stomatal closure and reduces water evaporation, which leads to enhanced drought tolerance [64]. Consistent with these reports, *slpip2;4* mutant plants in our study exhibited elevated ROS levels, decreased activities of SOD, CAT, and POD (Fig. 6), supporting a positive role of SlPIP2;4 in both redox balance and drought adaptation. Notably, although *slpip2;4* mutant plants displayed smaller stomatal apertures under drought conditions (Fig. S11), they exhibited higher gs and lower RWC than WT (Fig. 6k and l). These observations suggest that gs is not solely determined by aperture size and may also reflect other factors, such as whole-leaf hydraulic properties and plant water status. Future studies employing time-resolved gs measurements and ABA/ROS-induced stomatal closure kinetics would help further dissect the contribution of SlPIP2;4 to stomatal regulation and drought responses.

### Genetic evidence supports a hierarchical relationship between SlERF26 and SlPIP2;4

Genetic analysis using *SlERF26* OE × *slpip2;4* hybrid lines provided important insight into the relationship between SlERF26 and SlPIP2;4 in drought tolerance. The intermediate phenotypes observed in the hybrid plants, including survival rates, RWC, and ROS accumulation, indicate that the contribution of SlERF26 to drought tolerance is at least partially dependent on SlPIP2;4. Mechanistically, coordinated changes in ROS accumulation, antioxidant gene expression, and antioxidant enzyme activities across the different genotypes support a model in which SlERF26 functions upstream to modulate ROS homeostasis. On the one hand, SlERF26 activates SlPIP2;4 expression, which may influence H₂O₂ distribution and cellular redox balance. On the other hand, SlERF26 enhances antioxidant capacity at both the transcriptional and enzymatic levels, thereby promoting efficient ROS detoxification. Together, these findings support a framework in which SlERF26 integrates SlPIP2;4-mediated processes to fine-tune ROS homeostasis and improve drought tolerance.

The SlERF26-SlPIP2;4 regulatory module identified in this study complements current models of ERF-mediated drought responses by offering a more nuanced perspective on cellular redox homeostasis. Earlier studies have predominantly focused on ERF TFs directly regulating antioxidant enzyme genes [40] or modulating hormone signaling pathways, such as ABA-dependent responses [23, 33, 65, 66], to enhance stress tolerance. In these models, ERFs primarily function as transcriptional regulators that control downstream stress-responsive gene expression. In contrast, our findings reveal that SlERF26 not only participates in transcriptional regulation but also contributes to the regulation of intracellular H₂O₂ level, potentially through SlPIP2;4. This introduces an additional regulatory layer involving ROS transport, which is largely absent from conventional ERF-centered models. Notably, the partial retention of drought tolerance in the *SlERF26* OE × *slpip2;4* background further suggests that SlERF26 integrates multiple downstream pathways, including both ROS redistribution and ROS scavenging. Therefore, unlike the canonical ERF-antioxidant enzyme or ERF-hormone signaling modules, the SlERF26–SlPIP2;4 module represents a dual regulatory system that coordinates ROS transport and detoxification. Collectively, these observations extend current ERF-centered models by indicating a potential contribution of ROS transport, in addition to ROS detoxification, to drought-associated redox regulation.

Based on the molecular and genetic evidence, we propose that drought-induced *SlERF26* acts upstream of *SlPIP2;4* to coordinate redox homeostasis and drought adaptation in tomato. Under drought stress, the SlERF26 protein directly binds the DRE motif in the *SlPIP2;4* promoter and activates its transcription, thereby increasing SlPIP2;4 abundance at the plasma membrane. Elevated SlPIP2;4 may contribute to the regulation of intracellular H₂O₂ distribution and water status, while SlERF26 also enhances antioxidant capacity by upregulating ROS-detoxifying components at both the transcriptional and enzymatic levels. As a result, ROS accumulation and oxidative damage are restrained, and plants maintain improved water retention and survival under drought stress (Fig. 8).

**Figure 8:**
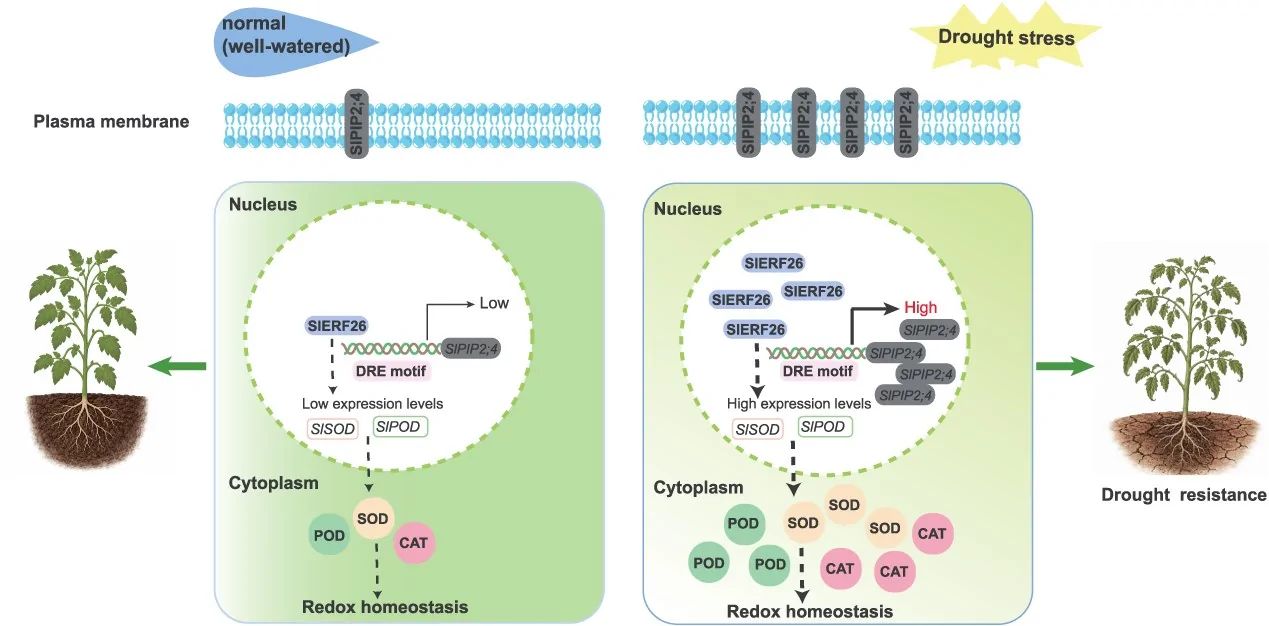
Proposed model of the SlERF26–SlPIP2;4 module in drought tolerance. Under well-watered (normal) conditions, basal expression of *SlERF26* maintains *SlPIP2;4* and redox status at homeostatic levels. Under drought stress, *SlERF26* is rapidly induced and accumulates in the nucleus, where it directly binds the DRE motif in the promoter of *SlPIP2;4* and activates its transcription. Increased *SlPIP2;4* at the plasma membrane may facilitate H₂O₂ movement across membranes and contribute to cellular redox balance and water status regulation. In parallel, SlERF26 enhances antioxidant capacity by promoting the expression (*SlPOD*, *SlSOD*) and activities of ROS-scavenging enzymes (SOD, POD, and CAT). Together, these coordinated processes reduce ROS accumulation and oxidative damage, improve water retention, and ultimately enhance drought tolerance.

## Materials and methods

### Gene isolation, vector construction, and generation of transgenic tomato

The *SlERF26* (SGN: Solyc06g035700) coding sequence was amplified from *S. lycopersicum* cv M82 using polymerase chain reaction (PCR). The full-length *SlERF26* PCR product was cloned into the pHellsgate8 vector [66], containing CaMV 35S promoter using homologous recombination following the manufacturer’s instructions (Hieff Clone® Plus, Yisheng, Shanghai, China). The recombinant plasmid was subsequently transformed into *A. tumefaciens* LBA4404 following the freeze–thaw technique for stable plant transformation.

The *SlERF26* OE tomato plants were generated using *Agrobacterium*-mediated transformation of tomato cotyledon explants as previously described [5]. Then, the transgenic lines were confirmed by PCR using gene-specific primers (Table S3), and their further expression was verified by RT-qPCR. To select for transgenic lines, plants were grown on Murashige and Skoog (MS) medium containing 50 mg/L kanamycin. To generate CRISPR/Cas9-mediated knockout of the *SlPIP2;4*, two guide RNAs (gRNAs) targeting the coding sequence were designed using the CRISPR-p v2.0 platform. The designed gRNAs were inserted into the pTX vector via homologous recombination [67]. The resulting CRISPR/Cas9 constructs, which utilize the U6 promoter for efficient expression in plants, were introduced into *A. tumefaciens* LBA4404 to mediate tomato transformation.

To evaluate the drought resistance of *SlERF26* OE lines and *SlPIP2;4* knockout lines in a single plant, we crossed *SlERF26* OE6 with *slpip2;4*-CR1 plants and *SlERF26*-OE18 with *slpip2;4*-CR2 plants to generate the OE6/CR1, and OE18/CR2 double mutants. PCR with primers specific for the SlERF26 overexpression construct and SlPIP2;4 CRISPR loci (Table S3) confirmed the presence of the target mutations. RT-qPCR was conducted to verify the transcription levels of *SlERF26* and *SlPIP2;4* in the hybrid plants.

### Bioinformatics analysis

The *SlERF26* gene sequence, along with the corresponding sequences of ERF26 homologs in 25 plant species were obtained from the NCBI database. These sequences were selected based on their homology to *SlERF26* and the availability of full-length coding sequences. The conserved protein motifs of SlERF26 and other ERF26 proteins were identified using the SMART tool, which predicts functional domains and conserved regions within protein sequences.

The amino acid sequences were aligned with GeneDoc (version 2.7), followed by manual inspection to ensure correct alignment of conserved regions. Regions with poor alignment were excluded from the analysis.

Phylogenetic relationships among ERF26 proteins were inferred in MEGA (version 7) using the Neighbor-Joining approach. The analysis was based on aligned protein sequences, evolutionary distances were calculated using the Poisson correction model, and branch support was evaluated with 1000 bootstrap replicates.

The transmembrane topology of SlPIP2;4 was predicted using the TMHMM server based on a hidden Markov model. Structural models of SlPIP2;4 WT and the CR2 mutant were generated using the AlphaFold server. The model with the highest predicted local distance difference test (pLDDT) score was selected for subsequent analyses. Structural visualization and comparison were performed using PyMOL (version 3.0).

### Subcellular localization

The *SlERF26* ORF was PCR-amplified using primers bearing BamHI and KpnI restriction sites to enable subsequent cloning into the expression vector. Then, the PCR product having the mentioned restriction site was digested and ligated into the pCAMBIA2300 vector. The recombinant construct was verified by sequencing to verify the correct insertion of the *SlERF26* sequence. The construct pCAMBIA2300-35S-EGFP-SlERF26 was designed to express *SlERF26* fused to enhanced green fluorescent protein (EGFP) driven by the CaMV 35S promoter.

An empty 35S-EGFP construct was prepared in parallel as a control. Both the *SlERF26*-EGFP fusion construct and the control 35S-EGFP were co-transfected into *N. benthamiana* epidermal cells via *Agrobacterium*-mediated transformation following standard protocol.

After 24 h, epidermal cells were stained with 1 mM 4′,6-diamidino-2-phenylindole (DAPI) (Solarbio, Beijing, China) for 1 h to label nuclei. EGFP and DAPI fluorescence was detected using a Zeiss laser scanning microscope (LSM) 780 confocal microscope (Carl Zeiss, Göttingen, Germany) with excitation at 515 and 405 nm, respectively, to investigate the subcellular localization of the SlERF26–EGFP fusion protein.

### RNA extraction and real time RT-qPCR analysis

Total RNA was extracted from the tomato tissues using RNAiso Plus reagent (TaKaRa, Dalian, China) according to the manufacturer’s instructions.

First-strand cDNA was synthesized from 1 μg of DNase I-treated RNA using the HiFiScript gDNA Removal RT MasterMix (CoWin, Taizhou, China). RT-qPCR was performed on a CFX96 Real-Time PCR Detection System (Bio-Rad, USA) with SYBR Green-based 2xSP qPCR Mix (Bioground, Chongqing, China). All reactions followed the manufacturer’s protocols.

*SlActin* gene was used as an internal control, and relative transcript levels were calculated using the 2^−ΔΔ*Ct*^ method.

### Drought stress and physiological assays

Tomato plants were grown in a greenhouse under a 16-h light/8-h dark photoperiod at 25 ± 2°C. At the five-leaf stage, drought stress was imposed by completely withholding irrigation. Seedlings were transplanted into individual plastic pots (9 cm diameter) containing a homogeneous substrate mixture of peat:vermiculite:perlite (3:1:1, v/v/v). Prior to drought treatment, all pots were saturated with water until drainage occurred, ensuring uniform soil water content at the start of the experiment. The soil weight of each pot was standardized before stress imposition to minimize variation in initial water availability among genotypes. After the drought treatment, plants were rewatered and allowed to recover for 3–4 days. Phenotypic responses were recorded, including visual symptoms such as wilting and leaf curling.

The MDA and proline contents were determined using methods described [68, 69]. For MDA quantification, plant samples were homogenized in TCA, and the resulting supernatant was reacted with thiobarbituric acid to form a color complex, which was measured spectrophotometrically. Proline levels were measured using acid–ninhydrin reaction and quantified by absorbance at 520 nm.

Leaf conductivity was measured according to the method [70]. Leaf disks were incubated in distilled water at 100°C for 30 min. The electrical conductivity was recorded before and after boiling the samples, and relative conductivity was calculated as a measure of cell membrane integrity under stress. For ROS and biochemical measurements, samples were collected when drought-sensitive plants first exhibited visible wilting symptoms but before irreversible tissue collapse. For survival assays, drought was extended until severe wilting was observed in most plants, followed by rewatering to assess recovery.

Chlorophyll concentration was assessed by measuring the absorption spectra of leaf extracts at 470, 649, and 665 nm using a spectrophotometer. The leaves (0.1 g) were soaked in 10 ml of 95% ethanol for 24 h in the dark.

POD, CAT, and SOD activities were determined following standard protocol [71]. Enzyme activities were measured by spectrophotometry, where POD activity was evaluated by the oxidation of guaiacol, CAT activity was determined based on the decomposition of hydrogen peroxide, and SOD activity was assessed by the inhibition of NBT reduction.

The stomatal density and quantity were measured on the abaxial (lower) epidermis of the third or fourth fully expanded leaf using a photomicroscope. Stomatal density was expressed as the number of stomata per square millimeter. To investigate the stomatal microstructure, mature, fully expanded leaves were harvested from drought-stressed plants on Day 15, and the leaf surface was observed using a scanning electron microscope (SU3500, Hitachi High-Technologies Corp., Tokyo, Japan). The analysis was conducted in triplicate, and six representative fields of view were captured per replicate to ensure consistency in the data.

Leaf RWC was determined following a standard protocol with minor modifications. Fresh weight (FW) of fully expanded leaves was measured immediately after sampling. The leaves were then floated on distilled water in the dark for 12 h at room temperature to obtain the turgid weight (TW). Subsequently, the samples were oven-dried at 80°C for 24 h to determine the dry weight (DW). RWC was calculated using the following formula: RWC = (FW − DW)/(TW − DW) * 100.

Stomatal conductance was measured using a portable porometer (LI-6400XTR, LI-COR, USA). Measurements were performed on the abaxial surface of fully expanded leaves between 9:00 and 11:00 a.m. under stable environmental conditions. For each plant, at least three leaves were measured, and three independent biological replicates were included. At least three biological replicates were included for each treatment.

Intracellular H₂O₂ levels were detected using the fluorescent probe H₂DCFDA (2′,7′-dichlorodihydrofluorescein diacetate). Leaf samples were incubated in 10 μM H₂DCFDA solution prepared in phosphate-buffered saline (PBS, pH 7.4). To facilitate dye penetration, samples were subjected to vacuum infiltration for 10 min, followed by incubation in the dark at room temperature for a total of 30 min.

After incubation, the samples were washed three times with PBS (5 min each) to remove excess dye. Fluorescence signals were observed using a fluorescence microscope (excitation: 488 nm, emission: 525 nm). Images were captured under identical settings for all samples. Fluorescence intensity was quantified using ImageJ software.

For each genotype and treatment, three independent biological replicates were used. Each biological replicate consisted of fully expanded leaves collected from three individual plants and pooled as one sample. All collected samples were immediately frozen in liquid nitrogen and stored at −80°C until further analysis.

### RNA-seq analysis

Leaves of WT and two representative *SlERF26* overexpression lines (OE15 and OE18), selected based on their expression levels and drought tolerance phenotypes, were subjected to 5-day drought stress, harvested, and immediately frozen in liquid nitrogen for subsequent RNA extraction. Well-watered plants served as a control. Total RNA was extracted using a standard RNA isolation protocol. Next-generation sequencing was performed by Novogene (Beijing, China) using the Illumina HiSeq2500 platform to obtain high-quality RNA-seq data.

Raw reads were filtered to remove adaptor sequences, poly-N reads, and low-quality reads. Clean reads were aligned to the tomato reference genome (SL2.50) using HISAT2 (v2.0.5) with default parameters, and only uniquely mapped reads were retained for downstream analysis.

Gene-level read counts were generated using featureCounts (v1.5.0-p3). Differential expression analysis was conducted in R using DESeq2 (v1.20.0) based on raw count matrices and a negative binomial distribution model. Two independent biological replicates were included for each condition. Data normalization was performed using the DESeq2 median-of-ratios method, and *P*-values were adjusted using the Benjamini–Hochberg procedure to control the false discovery rate (FDR). Genes with an adjusted *P*-value < 0.05 and |log2 fold change| ≥ 1 were considered differentially expressed.

PCA and sample-to-sample correlation analyses were performed using normalized count data to assess replicate consistency. Fragments per kilobase of transcript per million mapped reads (FPKM) values were calculated for visualization purposes only.

For functional annotation, the identified genes were compared against several public databases, including COG, KEGG, GO, NR, Swiss-Prot, and Pfam. Enriched pathways and functional terms were considered to be associated with drought stress responses in the transgenic lines.

To validate the RNA-seq results, the expression patterns of selected genes were further confirmed by qRT-PCR.

### Yeast one-hybrid and dual-luciferase reporter assays

The full-length *SlERF26* CDS was amplified and inserted into the pGADT7 to produce the effector construct for assessing binding to the DRE element. The promoter of *SlPIP2;4*, containing three tandem DRE element and their mutants, was synthesized and cloned into the pAbAi vector as the reporter construct. Both constructs were co-introduced into *Saccharomyces cerevisiae* Y1HGold using the LiAc/SS carrier DNA/PEG method. Yeast transformants were selected on SD/-Ura medium and then transferred to SD/-Leu medium containing aureobasidin A (AbA; Cat. CA2332G; Coolaber, Beijing, China) and incubated for 5 days to screen for protein–DNA interactions based on growth inhibition.

For the dual-luciferase assay, the SlERF26 full-length CDS was cloned into pGADT7 under the CaMV35S promoter, and the *SlPIP2;4* promoter was cloned into pGreen II 0800-LUC as the reporter. Agrobacterium GV3101 carrying the effector and reporter constructs was infiltrated into *N. benthamiana* leaves. After 48 h, fluorescence imaging was performed using a Fusion FX system (Vilber Lourmat, France). Fluorescence intensity was captured and quantified using ImageJ2 in the Fiji platform to assess the relative fluorescence signal [72]. Each assay was repeated three times, with at least three independent plants per replicate.

### Recombinant proteins and electrophoretic mobility shift assay

The full-length *SlERF26* CDS was amplified and inserted into the pET-30a, containing a 6x His tag for protein purification, and transformed into *Escherichia coli* Rosetta BL21 (DE3). Protein expression was induced with 0.1 mM isopropyl β-D-1-thiogalactopyranoside (IPTG) at 37°C for 12 h. After induction, cells were collected by centrifugation and sonicated in lysis buffer. The His-tagged SlERF26 protein was then purified using the His-tag Protein Purification Kit (Cat. P2226; Beyotime, Shanghai, China), following the manufacturer’s protocol for affinity chromatography. Purity and concentration were confirmed by sodium dodecyl sulfate-polyacrylamide gel electrophoresis (SDS-PAGE). For the EMSA, a probe containing the DRE box from the *SlPIP2;4* promoter was synthesized by Qingke Company (Beijing, China) and labeled with biotin. The probe was annealed to form double-stranded oligonucleotides. The binding assay was conducted using the Chemiluminescent EMSA Kit (Cat. GS009; Beyotime, Shanghai, China) according to the manufacturer’s protocol. Briefly, the reaction mixture contained 10 μg of the recombinant His-SlERF26 protein and the biotin-labeled DRE probe, incubated for 30 min at 25°C. For competition assays, a 100-fold excess of unlabeled competitor probe was added before the labeled probe. The DNA–protein complexes were resolved on a 6% native polyacrylamide gel and transferred to a nitrocellulose membrane. The biotin-labeled probes were detected using a Chemiluminescence Detection System (OI600, Bio-OI Technology Co., Ltd., Guangzhou, China) and the results were visualized using a chemiluminescent imaging system. Each assay was repeated three times to ensure reproducibility.

### Virus-induced gene silencing

*SlPIP2;4* was silenced in tomato using TRV-based VIGS vectors (pTRV1/2) [73]. Gene-specific cDNA fragments (Table S3) were amplified and cloned into pTRV2, sequence-verified, and then transformed into *Agrobacterium tumefaciens* GV3101.

For VIGS assays, Agrobacterium cultures harboring pTRV1 and pTRV2 constructs were mixed at a 1:1 ratio and infiltrated into the cotyledons of 2-week-old tomato seedlings using a needleless syringe. Plants infiltrated with empty pTRV2 vector were used as negative controls.

After infiltration, plants were maintained in the dark for 24 h and then transferred to normal growth conditions (25°C, 16-h light/8-h dark photoperiod). Gene silencing efficiency was assessed by RT-qPCR analysis of SlPIP2;4 expression levels at 2–3 weeks postinfiltration. Only plants showing significant reduction in transcript levels were used for subsequent experiments. The effectiveness of the VIGS system was confirmed using a TRV-PDS construct, which resulted in a characteristic photobleaching phenotype.

To minimize potential off-target effects, the silencing fragment was designed based on a gene-specific region with low sequence similarity to other tomato genes, as determined by BLAST analysis against the tomato genome available at the Sol Genomics Network.

### Statistical analysis

All data are presented as mean ± standard deviation (SD). Statistical analyses were performed using GraphPad Prism (version 9.4.0; GraphPad Software, San Diego, CA, USA). For multiple group comparisons, one-way or two-way analysis of variance followed by Tukey’s multiple comparison test was used as appropriate. Statistical significance in figures is indicated as ^*^P < 0.05; ^**^P < 0.01; ^***^P < 0.001; ^****^P < 0.0001. The number of biological replicates (*n*) is indicated in the figure legends. Biological replicates refer to independent plants unless otherwise stated.

## Supplementary Material

Web_Material_uhag215

## Data Availability

The RNA-Seq raw data have been deposited in the Sequence Read Archive database of NCBI (National Center for Biotechnology Information) under accession number PRJNA1380757. All other data supporting the findings of this study, including phenotypic measurements, fluorescence quantification, and experimental metadata, are available within the paper and its Supporting Information files. Additional materials and datasets can be obtained from the corresponding author upon reasonable request.

## References

[1] Zhu J . Abiotic stress signaling and responses in plants. Cell. 2016;167:313–24 10.1016/j.cell.2016.08.029 PMC5104190 27716505

[2] Zhang H, Zhu J, Gong Z. et al. Abiotic stress responses in plants. Nat Rev Genet. 2022;23:104–19 10.1038/s41576-021-00413-0 34561623

[3] Liu L, Tang C, Zhang Y. et al. The SnRK2.2–ZmHsf28–JAZ14/17 module regulates drought tolerance in maize. New Phytol. 2024;243:1515–31 10.1111/nph.20355 39686522

[4] Li J, Ouyang B, Wang T. et al. HyPRP1 gene suppressed by multiple stresses plays a negative role in abiotic stress tolerance in tomato. Front Plant Sci. 2016;7:967 10.3389/fpls.2016.00967 PMC4925714 27446190

[5] Wang L, Zhou Y, Ding Y. et al. Novel flavin-containing monooxygenase protein FMO1 interacts with CAT2 to negatively regulate drought tolerance through ROS homeostasis and ABA signaling pathway in tomato. Hortic Res. 2023;10:uhad037 10.1093/hr/uhad037 PMC10124749 37101513

[6] Qi J, Song CP, Wang B. et al. Reactive oxygen species signaling and stomatal movement in plant responses to drought stress and pathogen attack. J Integr Plant Biol. 2018;60:805–26 10.1111/jipb.12654 29660240

[7] Sun X, Wang P, Jia X. et al. Improvement of drought tolerance by overexpressing MdATG18a is mediated by a modified antioxidant system and activated autophagy in transgenic apple. Plant Biotechnol J. 2018;16:545–57 10.1111/pbi.12794 PMC5787838 28703378

[8] Thirumalaikumar VP, Devkar V, Mehterov N. et al. NAC transcription factor JUNGBRUNNEN1 enhances drought tolerance in tomato. Plant Biotechnol J. 2018;16:354–66 10.1111/pbi.12776 PMC5787828 28640975

[9] Chang Y, Fang Y, Liu J. et al. Stress-induced nuclear translocation of ONAC023 improves drought and heat tolerance through multiple processes in rice. Nat Commun. 2024;15:5877 10.1038/s41467-024-50229-9 PMC11245485 38997294

[10] Jin X, Chai Q, Liu C. et al. Cotton GhNAC4 promotes drought tolerance by regulating secondary cell wall biosynthesis and ribosomal protein homeostasis. Plant J. 2024;117:1052–68 10.1111/tpj.16538 37934782

[11] Zhang F, Pan Z, Han C. et al. Pyrus betulaefolia ERF3 interacts with HsfC1a to coordinately regulate aquaporin PIP1;4 and NCED4 for drought tolerance. Hortic Res. 2024;11:uhae090 10.1093/hr/uhae090 PMC11116902 38799129

[12] Xiang Y, Sun X, Bian X. et al. The transcription factor ZmNAC49 reduces stomatal density and improves drought tolerance in maize. J Exp Bot. 2020;72:1399–410 10.1093/jxb/eraa507 33130877

[13] Wang L, Cheng H, Wang Q. et al. CmRCD1 represses flowering by directly interacting with CmBBX8 in summer chrysanthemum. Hortic Res. 2021;8:79 10.1038/s41438-021-00516-z PMC8012346 33790241

[14] Sakuma Y, Liu Q, Dubouzet JG. et al. DNA-binding specificity of the ERF/AP2 domain of Arabidopsis DREBs, transcription factors involved in dehydration- and cold-inducible gene expression. Biochem Biophys Res Commun. 2002;290:998–1009 10.1006/bbrc.2001.6299 11798174

[15] Nakano T, Suzuki K, Fujimura T. et al. Genome-wide analysis of the ERF gene family in Arabidopsis and rice. Plant Physiol. 2006;140:411–32 10.1104/pp.105.073783 PMC1361313 16407444

[16] Sharma MK, Kumar R, Solanke AU. et al. Identification, phylogeny, and transcript profiling of ERF family genes during development and abiotic stress treatments in tomato. Mol Gen Genomics. 2010;284:455–75 10.1007/s00438-010-0580-1 20922546

[17] Franco-Zorrilla JM, López-Vidriero I, Carrasco JL. et al. DNA-binding specificities of plant transcription factors and their potential to define target genes. Proc Natl Acad Sci USA. 2014;111:2367–72 10.1073/pnas.1316278111 PMC3926073 24477691

[18] Tang M, Sun J, Liu Y. et al. Isolation and functional characterization of the JcERF gene, a putative AP2/EREBP domain-containing transcription factor, in the woody oil plant Jatropha curcas. Plant Mol Biol. 2007;63:419–28 10.1007/s11103-006-9098-7 17103014

[19] Xu ZS, Xia LQ, Chen M. et al. Isolation and molecular characterization of the Triticum aestivum L. ethylene-responsive factor 1 (TaERF1) that increases multiple stress tolerance. Plant Mol Biol. 2007;65:719–32 10.1007/s11103-007-9237-9 17874224

[20] Lee SY, Hwang EY, Seok HY. et al. Arabidopsis AtERF71/HRE2 functions as a transcriptional activator via cis-acting GCC box or DRE/CRT element and is involved in root development through regulation of root cell expansion. Plant Cell Rep. 2015;34:223–31 10.1007/s00299-014-1701-9 25344007

[21] Müller M, Munné-Bosch S. Ethylene response factors: a key regulatory hub in hormone and stress signaling. Plant Physiol. 2015;169:32–41 10.1104/pp.15.00677 PMC4577411 26103991

[22] Wang Y, Xia D, Li W. et al. Overexpression of a tomato AP2/ERF transcription factor SlERF.B1 increases sensitivity to salt and drought stresses. Sci Hortic. 2022;304:111332

[23] Wang Z, Zhao X, Ren Z. et al. ZmERF21 directly regulates hormone signaling and stress-responsive gene expression to influence drought tolerance in maize seedlings. Plant Cell Environ. 2022;45:312–28 10.1111/pce.14243 34873716

[24] Li Z, Tian Y, Xu J. et al. A tomato ERF transcription factor, SlERF84, confers enhanced tolerance to drought and salt stress but negatively regulates immunity against pseudomonas syringae pv. Tomato DC3000. Plant Physiol Biochem. 2018;132:683–95 10.1016/j.plaphy.2018.08.022 30146417

[25] Zhu Y, Liu Y, Zhou K. et al. Overexpression of ZmEREBP60 enhances drought tolerance in maize. J Plant Physiol. 2022;275:153763 10.1016/j.jplph.2022.153763 35839657

[26] Khan M, Dahro B, Wang Y. et al. The transcription factor ERF110 promotes cold tolerance by directly regulating sugar and sterol biosynthesis in citrus. Plant J. 2024;119:2385–401 10.1111/tpj.16925 38985498

[27] Sewelam N, Kazan K, Thomas-Hall SR. et al. Ethylene response factor 6 is a regulator of reactive oxygen species signaling in Arabidopsis. PLoS One. 2013;8:e70289 10.1371/journal.pone.0070289 PMC3734174 23940555

[28] Liu D, Chen X, Liu J. et al. The rice ERF transcription factor OsERF922 negatively regulates resistance to Magnaporthe oryzae and salt tolerance. J Exp Bot. 2012;63:3899–911 10.1093/jxb/ers079 PMC3388842 22442415

[29] Pan Y, Seymour GB, Lu C. et al. An ethylene response factor (ERF5) promotes adaptation to drought and salt tolerance in tomato. Plant Cell Rep. 2012;31:349–60 10.1007/s00299-011-1170-3 22038370

[30] Yao Y, He RJ, Xie QL. et al. ETHYLENE RESPONSE FACTOR 74 (ERF74) plays an essential role in controlling a respiratory burst oxidase homolog D (RbohD)-dependent mechanism in response to different stresses in Arabidopsis. New Phytol. 2017;213:1667–81 10.1111/nph.14278 28164334

[31] Yang R, Liu J, Lin Z. et al. ERF transcription factors involved in salt response in tomato. Plant Growth Regul. 2018;84:573–82

[32] Qu Y, Nong Q, Jian S. et al. An AP2/ERF gene, HuERF1, from pitaya (Hylocereus undatus) positively regulates salt tolerance. Int J Mol Sci. 2020;21:4586 10.3390/ijms21134586 PMC7369839 32605158

[33] Li N, Lu F, Kuai B. SlERF.D2-mediated antagonism between ethylene and ABA signaling pathways modulates osmotic stress adaptation in tomato. Hortic Res. 2025;13:uhaf267 10.1093/hr/uhaf267 PMC12861479 41631149

[34] Zhang H, Liu W, Wan L. et al. Functional analyses of ethylene response factor JERF3 with the aim of improving tolerance to drought and osmotic stress in transgenic rice. Transgenic Res. 2010;19:809–18 10.1007/s11248-009-9357-x 20087656

[35] Gong P, Zhang J, Li H. et al. Transcriptional profiles of drought-responsive genes in modulating transcription signal transduction, and biochemical pathways in tomato. J Exp Bot. 2010;61:3563–75 10.1093/jxb/erq167 PMC2921197 20643807

[36] Eshed Y, Abu-Abied M, Saranga Y. et al. Lycopersicon esculentum lines containing small overlapping introgressions from L. pennellii. Theor Appl Genet. 1992;83:1027–34 10.1007/BF00232968 24202931

[37] Zhang S, Feng M, Chen W. et al. In rose, transcription factor PTM balances growth and drought survival via PIP2;1 aquaporin. Nat Plants. 2019;5:290–9 10.1038/s41477-019-0376-1 30833710

[38] Cheng M, Liao PM, Kuo WW. et al. The Arabidopsis ETHYLENE RESPONSE FACTOR1 regulates abiotic stress-responsive gene expression by binding to different cis-acting elements in response to different stress signals. Plant Physiol. 2013;162:1566–82 10.1104/pp.113.221911 PMC3707555 23719892

[39] Zhang G, Chen M, Li L. et al. Overexpression of the soybean GmERF3 gene, an AP2/ERF type transcription factor for increased tolerances to salt, drought, and diseases in transgenic tobacco. J Exp Bot. 2009;60:3781–96 10.1093/jxb/erp214 PMC2736888 19602544

[40] Zhao Q, Hu RS, Liu D. et al. The AP2 transcription factor NtERF172 confers drought resistance by modifying NtCAT. Plant Biotechnol J. 2020;18:2444–55 10.1111/pbi.13419 PMC7680539 32445603

[41] Chen H, Shi Y, An L. et al. Overexpression of SlWRKY6 enhances drought tolerance by strengthening antioxidant defense and stomatal closure via ABA signaling in Solanum lycopersicum L. Plant Physiol Biochem. 2024;213:108855 10.1016/j.plaphy.2024.108855 38917736

[42] Gilbert ME, Medina V. Drought adaptation mechanisms should guide experimental design. Trends Plant Sci. 2016;21:639–47 10.1016/j.tplants.2016.03.003 27090148

[43] Hu N, Tang N, Yan F. et al. Effect of LeERF1 and LeERF2 overexpression in the response to salinity of young tomato (Solanum lycopersicum cv. Micro-tom) seedlings. Acta Physiol Plant. 2014;36:1703–12

[44] Upadhyay RK, Soni DK, Singh R. et al. SlERF36, an EAR-motif-containing ERF gene from tomato, alters stomatal density and modulates photosynthesis and growth. J Exp Bot. 2013;64:3237–47 10.1093/jxb/ert162 PMC3733148 23840010

[45] Muller B, Pantin F, Génard M. et al. Water deficits uncouple growth from photosynthesis, increase C content, and modify the relationships between C and growth in sink organs. J Exp Bot. 2011;62:1715–29 10.1093/jxb/erq438 21239376

[46] Bogeat-Triboulot M-BA, Brosché M, Renaut J. et al. Gradual soil water depletion results in reversible changes of gene expression, protein profiles, ecophysiology, and growth performance in Populus euphratica, a poplar growing in arid regions. Plant Physiol. 2007;143:876–92 10.1104/pp.106.088708 PMC1803728 17158588

[47] He X, Wang C, Wang H. et al. The function of MAPK cascades in response to various stresses in horticultural plants. Front Plant Sci. 2020;11:952 10.3389/fpls.2020.00952 PMC7412866 32849671

[48] Lin L, Wu J, Jiang M. et al. Plant mitogen-activated protein kinase cascades in environmental stresses. Int J Mol Sci. 2021;22:1543 10.3390/ijms22041543 PMC7913722 33546499

[49] Wang R, Li R, Cheng L. et al. SlERF52 regulates SlTIP1;1 expression to accelerate tomato pedicel abscission. Plant Physiol. 2021;185:1829–46 10.1093/plphys/kiab026 PMC8133580 33638643

[50] Zhang M, Shi H, Li N. et al. Aquaporin OsPIP2;2 links H₂O₂ signaling and transcriptional regulation. Plant Physiol. 2022;188:2325–41 10.1093/plphys/kiab604 PMC8968290 34958388

[51] Hakim , Ullah A, Hussain A. et al. Osmotin: a plant defense tool against stresses. Plant Physiol Biochem. 2018;123:149–59 10.1016/j.plaphy.2017.12.012 29245030

[52] Xiong H, Yu J, Miao J. et al. Natural variation in OsLG3 increases drought tolerance via ROS scavenging. Plant Physiol. 2018;178:451–67 10.1104/pp.17.01492 PMC6130013 30068540

[53] Cao J, Tan X. Comprehensive analysis of chitinase family genes in tomato. Plants. 2019;8:52 10.3390/plants8030052 PMC6473868 30823433

[54] Yu Z, Chen X, Chen Z. et al. BcSRC2 interacts with BcAPX4 to enhance drought tolerance via ABA signaling. Hortic Res. 2024;11:uhae165 10.1093/hr/uhae165 PMC11784589 39896045

[55] Xin X, Huang Z, Zhai T. et al. ZmChi7 confers salt tolerance via ROS homeostasis in maize. Plant Physiol Biochem. 2026;230:110906 10.1016/j.plaphy.2025.110906 41365124

[56] Kapilan R, Vaziri M, Zwiazek JJ. Regulation of aquaporins in plants under stress. Biol Res. 2018;51:4 10.1186/s40659-018-0152-0 PMC5769316 29338771

[57] Mittler R, Zandalinas SI, Fichman Y. et al. Reactive oxygen species signalling in plant stress responses. Nat Rev Mol Cell Biol. 2022;23:663–79 10.1038/s41580-022-00499-2 35760900

[58] Miao Y, Lv D, Wang P. et al. An Arabidopsis glutathione peroxidase functions as both a redox transducer and a scavenger in abscisic acid and drought stress responses. Plant Cell. 2006;18:2749–66 10.1105/tpc.106.044230 PMC1626619 16998070

[59] Grondin A, Rodrigues O, Verdoucq L. et al. Aquaporins contribute to ABA-triggered stomatal closure through OST1-mediated phosphorylation. Plant Cell. 2015;27:1945–54 10.1105/tpc.15.00421 PMC4531361 26163575

[60] Gong Z, Xiong L, Shi H. et al. Plant abiotic stress response and nutrient use efficiency. Sci China Life Sci. 2020;63:635–74 10.1007/s11427-020-1683-x 32246404

[61] Ahmed S, Kouser S, Asgher M. et al. Plant aquaporins: a frontward to make crop plants drought resistant. Physiol Plant. 2021;172:1089–105 10.1111/ppl.13416 33826759

[62] Zargar SM, Nagar P, Deshmukh R. et al. Aquaporins as potential drought tolerance inducing proteins: towards instigating stress tolerance. J Proteome. 2017;169:233–8 10.1016/j.jprot.2017.04.010 28412527

[63] Li R, Wang J, Li S. et al. Plasma membrane intrinsic proteins SlPIP2;1, SlPIP2;7 and SlPIP2;5 conferring enhanced drought stress tolerance in tomato. Sci Rep. 2016;6:31814 10.1038/srep31814 PMC4992886 27545827

[64] Xin H, Li Q, Wang S. et al. PIP2;4 improves growth and drought tolerance via stomatal regulation. Plant Sci. 2023;326:111526 10.1016/j.plantsci.2022.111526 36343868

[65] Wang J, Chen L, Long Y. et al. ZmHsf08 negatively regulates stress responses. Int J Mol Sci. 2021;22:11922 10.3390/ijms222111922 PMC8584904 34769354

[66] Song J, Shang L, Li C. et al. Variation in the fruit development gene POINTED TIP regulates protuberance of tomato fruit tip. Nat Commun. 2022;13:5940 10.1038/s41467-022-33648-4 PMC9547884 36209204

[67] Li J, Ai G, Wang Y. et al. A truncated B-box zinc finger transcription factor confers drought sensitivity in modern cultivated tomatoes. Nat Commun. 2024;15:8013 10.1038/s41467-024-51699-7 PMC11399245 39271661

[68] Hodges DM, DeLong JM, Forney CF. et al. Improving the thiobarbituric acid-reactive-substances assay for estimating lipid peroxidation in plant tissues containing anthocyanin and other interfering compounds. Planta. 1999;207:604–11 10.1007/s00425-017-2699-3 28456836

[69] Bates LS, Waldren RP, Teare ID. Rapid determination of free proline for water-stress studies. Plant Soil. 1973;39:205–7

[70] Blum A, Ebercon A. Cell membrane stability as a measure of drought and heat tolerance in wheat. Crop Sci. 1981;21:43–7

[71] Maehly AC, Chance B. The assay of catalases and peroxidases. Methods Biochem Anal. 1954;1:357–424 10.1002/9780470110171.ch14 13193536

[72] Hu N, Tian H, Li Y. et al. pHNRhCas9NG, single expression cassette-based dual-component dual-transcription unit CRISPR/Cas9 system for plant genome editing. Trends Biotechnol. 2025;43:1788–808 10.1016/j.tibtech.2025.03.016 40280813

[73] Liu Y, Schiff M, Dinesh-Kumar SP. Virus-induced gene silencing in tomato. Plant J. 2002;31:777–86 10.1046/j.1365-313x.2002.01394.x 12220268

