# Supplementary material for: Tomato SlERF26 enhances drought tolerance by regulating SlPIP2;4-mediated ROS homeostasis
Source: Hortic Res. 2026 Jun 2;13(11):uhag215. doi: 10.1093/hr/uhag215 (PMC13719176; doi:10.1093/hr/uhag215)
Supplement: Supplementary file 1 [file web_material_uhag215.zip › Supplementary_materials_uhag215.docx]

**Supplementary Figures**

**Figure S1.** Comparison of the -2000bp promoter sequence(a) and the coding sequence (b) of *SlERF26* between *S. lycopersicum* cv. M82 and *S. pennellii.*

**Figure S2. Amino acid alignment of ERF26s.** CLUSTAL alignment of deduced amino acid sequence of *Solanum lycopersicum* cv.M82(SlERF26) and the ERF sequence of *S. tuberosum* (StERF26), *S.verrucosum* (SvERF26), *S. stenotomum* (SsERF26), *Nicotiana tomentosiformis* (NtERF26), Capsicum baccatum (CbERF), C. annuum (CaERF26), *N. sylvestris* (NsERF26), *Camellia lanceoleosa* (ClERF26), *Striga hermonthica* (ShERF26), *Vitis vinifera* (VvERF26), *Olea europaea* var. sylvestris (OeERF26), *Daucus carota* subsp.sativus (DcERF26), *Cucurbita pepo* subsp.pepo (CpERF26), and *Rhodamnia argentea* (RaERF26). Red lines above the sequences represents the putative AP2 domain. Red triangles under the sequence represents the conserved alanine and aspartic acid residues in the AP2 domain.

**Figure S3. Phylogenetic tree depicting the relationships between SlERF26 and other ERF - related proteins from diverse species.** The alignments were carried out in MEGA 7.0 with the default parameters. Bootstrap values were replicated 1000 times.

**Figure S4. Analysis of *cis*-acting elements in the promoter region of the *SlERF26* gene.** Approximately 3.0 kb of the genomic DNA sequence upstream of the *SlERF26* coding region was isolated. Subsequently, putative *cis*-regulatory elements within this promoter region were analyzed using the PlantCARE database. Different colors are used to represent distinct *cis*-acting elements.

**Figure S5. SlERF26 positively regulates drought tolerance in tomato**

(a) Sequence alignment of the SlERF26 target sites in CRISPR/Cas9-generated mutant lines. The gRNA target sequences are indicated in red. The wild-type (WT) sequence is shown at the top, with mutation types for three independent knockout lines (CR-1, CR-2, and CR-3) listed below. For CR-1, a 32-bp deletion is observed at the target site. CR2 contains a 1-bp insertion of T and a 3-bp deletion. CR3 harbors a 4-bp deletion and another 3-bp deletion. Dashed lines (-) represent deleted bases, and plus sign (+) indicates inserted nucleotides.

(b) Phenotypic responses of *slerf26* mutant plants (CR-1, CR-2 and CR-3) and WT plants under drought conditions and rewatering. Plants were subjected to drought treatment for 5d. Representative images are shown. Scale bars= 10 cm.

(c, d) Tissue staining of WT and *slerf26* mutant plants under drought stress was conducted using NBT(c) and DAB(d). Drought tolerance assays were independently repeated three times, yielding consistent results, and a representative image is presented here. Scale bars= 1 cm.

**Figure S6. Sample-to-sample correlation and principal component analysis (PCA) of RNA-seq data.**

(a) Heatmap of Pearson correlation coefficients (R²) among samples based on gene expression profiles. Color intensity indicates correlation strength (light purple, low; dark blue, high). Samples are grouped by condition and genotype: CKWT, CKOE15, CKOE18, DTWT, DTOE15, and DTOE18, with numbers indicating biological replicates. Diagonal values are 1.0 by definition.

(b) PCA of RNA-seq samples based on DESeq2 rlog-transformed counts. PC1 (61.67%) and PC2 (12.83%) primarily separate samples by treatment (control vs. drought) and genotype (WT vs. OE). Each point represents an individual biological replicate.

**Figure S7. Histogram depicting the number of differentially expressed genes (DEG) between the wild-type (WT) tomato and *SlERF26* overexpressing lines (OE).** CKWT, CKOE-15, and CKOE-18 represent plants grown under normal conditions, while DTWT, DTOE-15, and DTOE -18 are those subjected to drought treatment. DEGs were identified through the comparison of transcriptomes between two samples.

**Figure S8. Validation of the transcriptional levels of 12 genes selected from the differentially expressed genes (DEGs).** Wild plants, as well as the OE15 and OE18 lines, were subjected to drought treatment, denoted as DTWT, DTOE15, and DTOE18. In the control group, a normal water supply was maintained, denoted as CKWT, CKOE15, and CKOE18. The selected DEGs include MYB and NAC transcription factors, UDP-glycosyltransferase (UDPGT), peroxidase, etc. Data represent the means of three biological replicates ± SD.

**Figure S9 Drought phenotypes of *SlPIP2,4*-silenced plants and the efficiency of *SlPIP2,4* gene silencing.** (a) Phenotypes of non-silenced (pTRV) and *SlPIP2,4*-silenced (pTRV - *SlPIP2,4*) tomato plants exposed to drought for 7 days. Scale bars= 10 cm. (b) Tissue staining of different lines under drought stress was conducted using NBT and DAB. Drought tolerance assays were independently performed three times, and similar results were obtained; a representative image is presented. Scale bars= 1 cm.(c) Photobleaching phenotype of TRV-PDS plants, indicating the effectiveness of the VIGS system. Scale bars= 5 cm. (d) Efficiency of *SlPIP2,4* gene silencing by virus-induced gene silencing (VIGS). Data are presented as the means of three biological replicates (±SD). Asterisks indicate significant differences (*****p* < 0.0001, Student’s t test).

**Figure S10.** **Sequence, structural, and mutational characterization of SlPIP2;4 and its CRISPR-derived variants**

(a–c) Targeted mutations in *SlPIP2;4* generated by CRISPR–Cas9. Amino acid sequences surrounding the sgRNA target sites are shown for WT and mutant lines.

(d) Multiple sequence alignment of PIP2 aquaporins from Arabidopsis, maize, rice, potato, and tomato. The transmembrane helices and loop regions were predicted for SlPIP2;4 using TMHMM.

(e, f) Structural comparison between SlPIP2;4 (WT) and the CR2 mutant modeled by AlphaFold. WT is shown in green and CR2 in cyan. Regions showing structural differences between WT and CR2 are highlighted in red (WT) and yellow (CR2), including Loop A (e) and TM3 with Loop C (f).

**Figure S11.** **Stomatal traits and responsiveness of slpip2;4 mutants under drought conditions.**

(a) Stomatal microstructure images of slpip2;4 mutant plants and WT plants under drought conditions were obtained. (b) Analysis of stomatal density and (c) stomatal aperture (width/length) was conducted on slpip2;4 mutant plants and the WT plants. Data are presented as mean ± SD from three biological replicates. Statistical significance was determined by one-way ANOVA followed by Tukey’s multiple comparison test. Asterisks indicate significant differences compared to wild type (WT): *p < 0.05, ****p < 0.0001.

**Figure S12.** ***SlPIP2;4* regulates ROS accumulation under drought stress.**

(a) Survival rate was determined in tomato seedlings of different lines under control (grey bars) and drought (orange bars) condition. Data are presented as mean ± SD from three independent biological replicates. Statistical significance was determined by one-way ANOVA followed by Tukey’s multiple comparison test. Asterisks indicate significant differences compared to WT: ****p < 0.0001.

(b) RWC of different lines under control and drought conditions.

(c, d) Relative expression levels of antioxidant enzyme genes *POD* and *SOD* in different lines under control (grey bars) and drought (orange bars) conditions.

Data are presented as mean ± SD from three independent biological replicates. Statistical significance was determined by two-way ANOVA followed by Tukey’s multiple comparison test. Asterisks indicate significant differences compared to WT: *p < 0.05, **p < 0.01, ***p < 0.001, ****p < 0.0001.

(e, f) Tissue staining of different lines under drought stress was conducted using NBT(e) and DAB(f). Drought tolerance assays were independently performed three times, and similar results were obtained; a representative image is presented. Scale bars= 1 cm.

**Supplementary Tables**

**Table S1 Summary of transcriptome sequencing data from leaves of tomato**

| **sample** | **raw_reads** | **clean_reads** | **clean_bases** | **error_rate** | **Q20** | **Q30** | **GC_pct** |
| --- | --- | --- | --- | --- | --- | --- | --- |
| CKOE18_1 | 47894678 | 46671694 | 7.0G | 0.03 | 97.22 | 91.96 | 42.57 |
| CKOE18_2 | 48025784 | 46244362 | 6.94G | 0.03 | 97.44 | 92.6 | 42.6 |
| CKOE15_1 | 47421748 | 46149098 | 6.92G | 0.03 | 97.25 | 92.06 | 42.73 |
| CKOE15_2 | 45495986 | 43264380 | 6.49G | 0.03 | 97.26 | 92.12 | 42.75 |
| CKWT_1 | 47048548 | 45763854 | 6.86G | 0.03 | 97.19 | 91.86 | 42.39 |
| CKWT_2 | 46467226 | 45014034 | 6.75G | 0.03 | 97.36 | 92.27 | 42.35 |
| DTOE18_1 | 47302994 | 45691318 | 6.85G | 0.03 | 97.24 | 92.04 | 42.26 |
| DTOE18_2 | 46361948 | 45068292 | 6.76G | 0.03 | 97.26 | 92.07 | 42.25 |
| DTOE15_1 | 41289820 | 40405138 | 6.06G | 0.03 | 97.15 | 91.79 | 42.2 |
| DTOE15_2 | 43714882 | 42310812 | 6.35G | 0.03 | 97.35 | 92.28 | 42.53 |
| DTWT_1 | 41313284 | 40706576 | 6.11G | 0.03 | 97.28 | 92.2 | 41.53 |
| DTWT_2 | 45131566 | 43528574 | 6.53G | 0.03 | 97.2 | 91.94 | 41.96 |

raw_reads: number of reads in original data; clean_reads: number of reads filtered in original data; clean_bases: number of bases filtered in original data (clean base=clean reads*150bp); error_rate: overall sequencing error rate of data; percentage of bases with Q20: the proportion of the nucleotide quality value greater than 20 in total bases; percentage of bases with Q30: the proportion of the nucleotide quality value larger than 30 in total bases. GC_pct: proportion of guanidine and cytosine nucleotides among the total nucleotides.

**Table S2 statistics of comparison between samples and reference genome**

| **sample** | **total_reads** | **total_map** | **unique_map** | **multi_map** |
| --- | --- | --- | --- | --- |
| CKOE18_1 | 46671694 | 44772058(95.93%) | 44052274(94.39%) | 719784(1.54%) |
| CKOE18_2 | 46244362 | 44480011(96.18%) | 43739885(94.58%) | 740126(1.6%) |
| CKOE15_1 | 46149098 | 43374033(93.99%) | 42717429(92.56%) | 656604(1.42%) |
| CKOE15_2 | 43264380 | 41480941(95.88%) | 40865339(94.45%) | 615602(1.42%) |
| CKWT_1 | 45763854 | 43900613(95.93%) | 43243924(94.49%) | 656689(1.43%) |
| CKWT_2 | 45014034 | 43278268(96.14%) | 42593947(94.62%) | 684321(1.52%) |
| DTOE18_1 | 45691318 | 43807741(95.88%) | 43203148(94.55%) | 604593(1.32%) |
| DTOE18_2 | 45068292 | 43142110(95.73%) | 42534601(94.38%) | 607509(1.35%) |
| DTOE15_1 | 40405138 | 38591290(95.51%) | 38062699(94.2%) | 528591(1.31%) |
| DTOE15_2 | 42310812 | 40003805(94.55%) | 39435437(93.2%) | 568368(1.34%) |
| DTWT_1 | 40706576 | 39112614(96.08%) | 38582032(94.78%) | 530582(1.3%) |
| DTWT_2 | 43528574 | 41804590(96.04%) | 41205664(94.66%) | 598926(1.38%) |

sample: sample name; total_reads: the number of clean reads of the sequencing data after quality control; total_map: the number and percentage of reads aligned to the genome; unique_map: the number and percentage of reads aligned to the unique location of the reference genome (for subsequent quantitative data analysis reads); multi_map: the number and percentage of reads aligned to multiple locations of the reference genome.

**Table S3. List of primers used in this study**

| **Primer name** | **Sequence** | **experiment** |
| --- | --- | --- |
| ERF26-OE-Fw | TAACCAACCCACGACTTTCC | Construction overexpression vector |
| ERF26-OE-Rv | CCGAAACAAACAGCATAACAAA |  |
| ERF26-Q-Fw | GTTGCAGCTTTGGCATTAAAAG | QRT-PCR primers |
| ERF26-Q-Rv | CTCCTGCTGCTGTATTGATTTC |  |
| ERF26-GFP-Fw | agggtacccggggatcctctagaATGGCTGCTTATCATTTTAATGACA | Construction of subcellular localization vector |
| ERF26-GFP-Rv | cttgctcaccatggtactagtTGGATAACCCCAGAGACTATCTG |  |
| PIP2,4-DRE1-Fw | AGCTTTACCGACATTACCGACATTACCGACATC | Construction of Y1H vector |
| PIP2,4-DRE1-Rv | TCGAGATGTCGGTAATGTCGGTAATGTCGGTAA |  |
| PIP2,4-DRE-M2-Fw | AGCTTTACGGCCATTACGGCCATTACGGCCATC |  |
| PIP2,4-DRE-M2-Rv | TCGAGATGGCCGTAATGGCCGTAATGGCCGTAA |  |
| ERF26-AD-Fw | GTACCAGATTACGCTCATATGATGGCTGCTTATCATTTTAATGACA |  |
| ERF26-AD-Rv | ACGATTCATCTGCAGCTCGAGTGGATAACCCCAGAGACTATCTG |  |
| PIP2,4-0800-Fw | cccAAGCTTTGCTGTCATTGGTAGGTTTA | Construction of LUC/REN |
| PIP2,4-0800-Rv | TTCTGCAGGAACCACATAAACAAAAGTTTT |  |
| ERF26-SK-Fw | aggacagcccaagctgagctcATGGCTGCTTATCATTTTAATGACA |  |
| ERF26-SK-Rv | gtcgacggtatcgataagcttTCATGGATAACCCCAGAGACT |  |
| PIP2,4-MA-Fw | CAGCAGTGACCTTTGGGCTA | Vector detection primer |
| PIP2,4-MA-Rv | TCCTTGTTGGATCTATGGTCGAC |  |
| PIP2,4-CR-Fw | ATATATGGTCTCGTTTGCTATACTAGGGGCGTTGCAGTTTTAGAGCTAGAAATAGC | Construction of knockout Vector |
| PIP2,4-CR-Rv | ATTATTGGTCTCGAAACAGAAGCCCAACACTACCACATGCACCAGCCGGGAATCGAA |  |
| PIP2,4-EMSA-Fw | CATGTCATATTTTTGTCGGGATATACTCTATTCA | Construction of EMSA Vector |
| PIP2,4-EMSA-Rv | TGAATAGAGTATATCCCGACAAAAATATGACATG |  |
| ERF26-30a-Fw | GCTGATATCGGATCCGAATTCATGGCTGCTTATCATTTTAAT |  |
| ERF26-30a-Rv | GTGGTGGTGGTGGTGCTCGAGTGGATAACCCCAGAGACTATC |  |
| ACtin-F | GTCCTCTTCCAGCCATCCAT | Internal reference primers |
| ACtin-R | ACCACTGAGCACAATGTTACCG |  |
| AD-R | AGATGGTGCACGATGCACAG | Vector detection primer |
| T7-F | TAATACGACTCACTATAGGGC | Vector detection primer |
| 35S | ACGCACAATCCCACTATCCTTC | Vector detection primer |
| PIP2,4-pDRE-Fw | TGTCGGTGTCGGTGTCGGTGTCGGTGTCGG | EMSA probe |
| PIP2,4-pDRE-Rv | CCGACACCGACACCGACACCGACACCGACA |  |
| UDPGT-Q-Fw | TAGCTTGGGGTCTCGAACTG | RT-qPCR primers |
| UDPGT-Q-R | TTTCATCGCTTCCCACGTTG |  |
| ABA hydroxylase 3-Q-Fw | ACAACGTTGCATTGCTTTCG |  |
| ABA hydroxylase 3-Q-R | TTTGCTAGCTCCTTTCGTGC |  |
| peroxidase 42-Q-Fw | GGGCCACACATTCCTCTCAA |  |
| peroxidase 42-Q-R | TCACACAGTGTGTTCGACCC |  |
| U-box 21-like-Q-Fw | TGCATAGTGGAATGTGGGGT |  |
| U-box 21-like-Q-Rv | CGTCATCGCCGATAACAAGT |  |
| NAC2-Q-Fw | TTGACAGTGGCTTTTGTGCT |  |
| NAC2-Q-Rv | TGCCTTCCAATACCCTGCTT |  |
| DIG3-Q-Fw | GGGAAAATCGCATCACACCA |  |
| DIG3-Q-Rv | AATCTCGTGGCTCAAATGGC |  |
| PYR1-Q-Fw | GTTACGACGGTGCATGGATT |  |
| PYR1-Q-Rv | GGCGTGTATCTTCCTCCGTA |  |
| PIP2-4-Q-Fw | CGGCGGAGCTAAAGAAATGGG |  |
| PIP2-4-Q-Rv | AAACCATGCCACCAAAGACC |  |
| aquaporin-5-Q-Fw | CCCAAATGGGCCCGTTATTGT |  |
| aquaporin-5-Q-Rv | CCCCTCCATAGCCCTTTCTC |  |
| PIP2-6-Q-Fw | TGCCACTATTCCGATCACCG |  |
| PIP2-6-Q-Rv | GCTTTAATTGCTCCGGCTCG |  |
